# Acceleration of the PDHGM on Partially Strongly Convex Functions

**DOI:** 10.1007/s10851-016-0692-2

**Published:** 2016-12-15

**Authors:** Tuomo Valkonen, Thomas Pock

**Affiliations:** 1 Department of Applied Mathematics and Theoretical Physics, University of Cambridge, Cambridge, UK; 2 Department of Mathematical Sciences, University of Liverpool, Liverpool, UK; 3 Institute for Computer Graphics and Vision, Graz University of Technology, 8010 Graz, Austria; 4 Digital Safety and Security Department, AIT Austrian Institute of Technology GmbH, 1220 Vienna, Austria

**Keywords:** Primal–dual, Accelerated, Subspace, Total generalised variation, 90C25, 49M29, 94A08

## Abstract

We propose several variants of the primal–dual method due to Chambolle and Pock. Without requiring full strong convexity of the objective functions, our methods are accelerated on subspaces with strong convexity. This yields mixed rates, $O(1{/}N^2)$ with respect to initialisation and *O*(1 / *N*) with respect to the dual sequence, and the residual part of the primal sequence. We demonstrate the efficacy of the proposed methods on image processing problems lacking strong convexity, such as total generalised variation denoising and total variation deblurring.

## Introduction

Let $G: X \rightarrow \overline{\mathbb {R}}$ and $F: Y \rightarrow \overline{\mathbb {R}}$ be convex, proper, and lower semicontinuous functionals on Hilbert spaces *X* and *Y*, possibly infinite dimensional. Also let $K \in \mathcal {L}(X; Y)$ be a bounded linear operator. We then wish to solve the problem

$$\begin{aligned} \min _{x \in X} G(x) + F(Kx). \end{aligned}$$

This can under mild conditions on *F* (see, for example, [1, 2]) also be written with the help of the convex conjugate $F^*$ in the minimax form

$$\begin{aligned} \min _{x \in X} \max _{y \in Y} \ G(x) + \langle K x,y\rangle - F^*(y). \end{aligned}$$

One possibility for the numerical solution of the latter form is the primal–dual algorithm of Chambolle and Pock [3], a type of proximal point or extragradient method, also classified as the ‘modified primal–dual hybrid gradient method’ or PDHGM by Esser et al. [4]. If either *G* or $F^*$ is strongly convex, the method can be accelerated to $O(1/N^2)$ convergence rates of the iterates and an ergodic duality gap [3]. But what if we have only partial strong convexity? For example, what if

$$\begin{aligned} G(x)=G_0(P x) \end{aligned}$$

for a projection operator *P* to a subspace $X_0 \subset X$, and strongly convex $G_0: X_0 \rightarrow \mathbb {R}$? This kind of structure is common in many applications in image processing and data science, as we will more closely review in Sect. 5. Under such *partial strong convexity*, can we obtain a method that would give an accelerated rate of convergence at least for *Px*?

We provide a partially positive answer: we can obtain mixed rates, $O(1/N^2)$ with respect to initialisation, and *O*(1 / *N*) with respect to bounds on the ‘residual variables’ *y* and $(I-P)x$. In this respect, our results are similar to the ‘optimal’ algorithm of Chen et al. [5]. Instead of strong convexity, they assume smoothness of *G* to derive a primal–dual algorithm based on backward–forward steps, instead of the backward–backward steps of [3].

The derivation of our algorithms is based, firstly, on replacing simple step length parameters by a variety of abstract step length operators and, secondly, a type of abstract partial strong monotonicity property

1 $$\begin{aligned}&\langle \partial G(x') - \partial G(x),{\widetilde{T}}^{-1}(x'-x)\rangle \nonumber \\&\quad \ge \Vert x'-x\Vert _{{{\widetilde{T}}}^{-1,*}\Gamma '}^2 - \text {penalty}\_\text {term}, \end{aligned}$$

the full details of which we provide in Sect. 2. Here ${\widetilde{T}}$ is an auxiliary step length operator. Our factor of strong convexity is a positive semidefinite operator $\Gamma \ge 0$; however, to make our algorithms work, we need to introduce additional artificial strong convexity through another operator $\Gamma '$, which may not satisfy $0 \le \Gamma ' \le \Gamma$. This introduces the penalty term in (1). The exact procedure can be seen as a type of smoothing, famously studied by Nesterov [6], and more recently, for instance, by Beck and Teboulle [7]. In these approaches, one computes *a priori* a level of smoothing—comparable to $\Gamma '$—needed to achieve prescribed solution quality. One then solves a smoothed problem, which can be done at $O(1/N^2)$ rate. However, to obtain a solution with higher quality than the a priori prescribed one, one needs to solve a new problem from scratch, as the smoothing alters the problem being solved. One can also employ restarting strategies, to take some advantage of the previous solution, see, for example, [8]. Our approach does not depend on restarting and a priori chosen solution qualities: the method will converge to an optimal solution to the original non-smooth problem. Indeed, the introduced additional strong convexity $\Gamma '$ is controlled automatically.

The ‘fast dual proximal gradient method’, or FDPG [9], also possesses different type of mixed rates, *O*(1 / *N*) for the primal, and $O(1/N^2)$ for the dual. This is, however, under standard strong convexity assumptions. Other than that, our work is related to various further developments from the PDHGM, such as variants for nonlinear *K* [10, 11] and non-convex *G* [12]. The PDHGM has been the basis for inertial methods for monotone inclusions [13] and primal–dual stochastic coordinate descent methods without separability requirements [14]. Finally, the FISTA [15, 16] can be seen as a primal-only relative of the PDHGM. Not attempting to do full justice here to the large family of closely related methods, we point to [4, 17, 18] for further references.

The contributions of our paper are twofold: firstly, to paint a bigger picture of what is possible, we derive a very general version of the PDHGM. This algorithm, useful as a basis for deriving other new algorithms besides ours, is the content of Sect. 2. In this section, we provide an abstract bound on the iterates of the algorithm, later used to derive convergence rates. In Sect. 3, we extend the bound to include an ergodic duality gap under stricter conditions on the acceleration scheme and the step length operators. A by-product of this work is the shortest convergence rate proof for the accelerated PDHGM known to us. Afterwards, in Sect. 4, we derive from the general algorithm two efficient mixed-rate algorithms for problems exhibiting strong convexity only on subspaces. The first one employs the penalty or smoothing $\psi$ on both the primal and the dual. The second one only employs the penalty on the dual. We finish the study with numerical experiments in Sect. 5. The main results of interest for readers wishing to apply our work are Algorithms 3 and 4 along with the respective convergence results, Theorems 4.1 and 4.2.

## A General Primal–Dual Method

### Notation

To make the notation definite, we denote by $\mathcal {L}(X; Y)$ the space of bounded linear operators between Hilbert spaces *X* and *Y*. For $T, S \in \mathcal {L}(X; X)$, the notation $T \ge S$ means that $T-S$ is positive semidefinite; in particular, $T \ge 0$ means that *T* is positive semidefinite. In this case, we also denote

$$\begin{aligned} {[0, T]} :=\{\lambda T \mid \lambda \in [0, 1]\}. \end{aligned}$$

The identity operator is denoted by *I*, as is standard.

For $0 \le M \in \mathcal {L}(X; X)$, which can possibly not be self-adjoint, we employ the notation

2 $$\begin{aligned} \langle a,b\rangle _M :=\langle Ma,b\rangle , \quad \text {and} \quad \Vert a\Vert _M :=\sqrt{\langle a,a\rangle _M}. \end{aligned}$$

We also use the notation ${T}^{-1,*} :=(T^{-1})^*$.

### Background

As in the introduction, let us be given convex, proper, lower semicontinuous functionals $G: X \rightarrow \overline{\mathbb {R}}$ and $F^*: Y \rightarrow \overline{\mathbb {R}}$ on Hilbert spaces *X* and *Y*, as well as a bounded linear operator $K \in \mathcal {L}(X; Y)$. We then wish to solve the minimax problem

P $$\begin{aligned} \min _{x \in X} \max _{y \in Y} \ G(x) + \langle K x,y\rangle - F^*(y), \end{aligned}$$

assuming the existence of a solution ${\widehat{u}}=({\widehat{x}}, {\widehat{y}})$ satisfying the optimality conditions

OC $$\begin{aligned} -K^* {\widehat{y}}\in \partial G({\widehat{x}}), \quad \text {and}\quad K {\widehat{x}}\in \partial F^*({\widehat{y}}). \end{aligned}$$

Such a point always exists if $\lim _{\Vert x\Vert \rightarrow \infty } G(x)/\Vert x\Vert =\infty$ and $\lim _{\Vert y\Vert \rightarrow \infty } F^*(y)/\Vert y\Vert =\infty$, as follows from [2, Proposition VI.1.2 & Proposition VI.2.2]. More generally the existence has to be proved explicitly. In finite dimensions, see, for example, [19] for several sufficient conditions.

The primal–dual method of Chambolle and Pock [3] for the solving (P) consists of iterating

3a $$\begin{aligned} x^{i+1}&:=(I+\tau _i \partial G)^{-1}(x^i- \tau _i K^*y^{i}), \end{aligned}$$

3b $$\begin{aligned} \bar{x}^{i+1}&:=\omega _i (x^{i+1}-x^i)+x^{i+1}, \end{aligned}$$

3c $$\begin{aligned} y^{i+1}&:=(I+\sigma _{i+1} \partial F^*)^{-1}(y^i+ \sigma _{i+1} K \bar{x}^{i+1}). \end{aligned}$$

In the basic version of the algorithm, $\omega _i=1$, $\tau _i \equiv \tau _0$, and $\sigma _i \equiv \sigma _0$, assuming that the step length parameters satisfy $\tau _0 \sigma _0 \Vert K\Vert ^2 < 1$. The method has *O*(1 / *N*) rate for the ergodic duality gap [3]. If *G* is strongly convex with factor $\gamma$, we may use the acceleration scheme [3]

4 $$\begin{aligned} \omega _i :=1/\sqrt{1+2\gamma \tau _i}, \quad \tau _{i+1} :=\tau _i\omega _i, \quad \text {and}\quad \sigma _{i+1} :=\sigma _i/\omega _i,\nonumber \\ \end{aligned}$$

to achieve $O(1/N^2)$ convergence rates of the iterates and an ergodic duality gap, defined in [3]. To motivate our choices later on, observe that $\sigma _0$ is never used expect to calculate $\sigma _1$. We may therefore equivalently parametrise the algorithm by $\delta = 1 - \Vert K\Vert ^2 \tau _0\sigma _0 > 0$.

We note that the order of the steps in (3) is different from the original ordering in [3]. This is because with the present order, the method (3) may also be written in the proximal point form. This formulation, first observed in [20] and later utilised in [10, 11, 21], is also what we will use to streamline our analysis. Introducing the general variable splitting notation,

$$\begin{aligned} u=(x, y), \end{aligned}$$

the system (3) then reduces into

5 $$\begin{aligned} 0 \in H(u^{i+1}) + M_{\text {basic},i}(u^{i+1}-u^i), \end{aligned}$$

for the monotone operator

6 $$\begin{aligned} H(u) :=\begin{pmatrix} \partial G(x) + K^* y \\ \partial F^*(y) -K x \end{pmatrix}, \end{aligned}$$

and the *preconditioning* or *step length operator*

7 $$\begin{aligned} M_{\text {basic},i} :=\begin{pmatrix} I/\tau _i &{}\quad -K^* \\ -\omega _i K &{}\quad I/\sigma _{i+1} \end{pmatrix}. \end{aligned}$$

We note that the optimality conditions (OC) can also be encoded as $0 \in H({\widehat{u}})$.

### Abstract Partial Monotonicity

Our plan now is to formulate a general version of (3), replacing $\tau _i$ and $\sigma _i$ by operators $T_i \in \mathcal {L}(X; X)$ and $\Sigma _i \in \mathcal {L}(Y; Y)$. In fact, we will need two additional operators ${\widetilde{T}}_i \in \mathcal {L}(X; X)$ and ${\hat{T}}_i \in \mathcal {L}(Y; Y)$ to help communicate change in $T_i$ to $\Sigma _i$. They replace $\omega _i$ in (3b) and (7), operating as ${\hat{T}}_{i+1} K {\widetilde{T}}^{-1}_i \approx \omega _i K$ from both sides of *K*. The role of ${\widetilde{T}}_i$ is to split the original primal step length $\tau _i$ in the space *X* into the two parts $T_i$ and ${\widetilde{T}}_i$ with potentially different rates. The role of ${\hat{T}}_i$ is to transfer ${\widetilde{T}}_i$ into the space *Y*, to eventually control the dual step length $\Sigma _i$. In the basic algorithm (3), we would simply have ${\widetilde{T}}_i=T_i=\tau _i I \in \mathcal {L}(X; X)$, and ${\hat{T}}_i=\tau _i I \in \mathcal {L}(Y; Y)$ for the scalar $\tau _i$.

To start the algorithm derivation, we now formulate abstract forms of partially strong monotonicity. As a first step, we take subsets of invertible operators

$$\begin{aligned} \widetilde{\mathcal {T}}\subset \mathcal {L}(X; X), \quad \text {and}\quad \hat{\mathcal {T}}\subset \mathcal {L}(Y; Y), \end{aligned}$$

as well as subsets of positive semidefinite operators

$$\begin{aligned} 0 \le \widetilde{\mathcal {K}}\subset \mathcal {L}(X; X), \quad \text {and}\quad 0 \le \hat{\mathcal {K}}\subset \mathcal {L}(Y; Y). \end{aligned}$$

We assume $\widetilde{\mathcal {T}}$ and $\hat{\mathcal {T}}$ closed with respect to composition: ${\widetilde{T}}_1{\widetilde{T}}_2 \in \widetilde{\mathcal {T}}$ for ${\widetilde{T}}_1,{\widetilde{T}}_2 \in \widetilde{\mathcal {T}}$.

We use the sets $\widetilde{\mathcal {K}}$ and $\hat{\mathcal {K}}$ as follows. We suppose that $\partial G$ is *partially strongly* $(\psi , \widetilde{\mathcal {T}}, \widetilde{\mathcal {K}})$ *-monotone*, meaning that for all $x, x' \in X, {\widetilde{T}}\in \widetilde{\mathcal {T}}$, $\Gamma ' \in [0, \Gamma ]+\widetilde{\mathcal {K}}$ holds

G-PM $$\begin{aligned}&\langle \partial G(x') - \partial G(x),{\widetilde{T}}^{-1}(x'-x)\rangle \nonumber \\&\quad \ge \Vert x'-x\Vert _{{{\widetilde{T}}}^{-1,*}\Gamma '}^2-\psi _{{{\widetilde{T}}}^{-1,*}(\Gamma '-\Gamma )}({x'-x}), \end{aligned}$$

for some family of functionals $\{\psi _{T}: X \rightarrow \mathbb {R}\}$, and a linear operator $0 \le \Gamma \in \mathcal {L}(X; X)$ which models partial strong monotonicity. The inequality in (G-PM), and all such set inequalities in the remainder of this paper, is understood to hold for all elements of the sets $\partial G(x')$ and $\partial G(x)$. The operator ${\widetilde{T}}\in \widetilde{\mathcal {T}}$ acts as a *testing operator*, and the operator $\Gamma ' \in \widetilde{\mathcal {K}}$ as *introduced strong monotonicity*. The functional $\psi _{{{\widetilde{T}}}^{-1,*}(\Gamma '-\Gamma )}$ is a *penalty* corresponding to the test and the introduced strong monotonicity. The role of testing will become more apparent in Sect. 2.4.

Similarly to (G-PM), we assume that $\partial F^*$ is $(\phi , \hat{\mathcal {T}}, \hat{\mathcal {K}})$ *-monotone* with respect to $\hat{\mathcal {T}}$ in the sense that for all $y, y' \in Y$, ${\hat{T}}\in \hat{\mathcal {T}}, R \in \hat{\mathcal {K}}$ holds 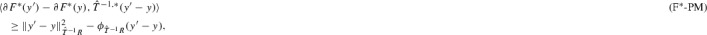


for some family of functionals $\{\phi _{T}: Y \rightarrow \mathbb {R}\}$. Again, the inequality in ($\hbox {F}^*$-PM) is understood to hold for all elements of the sets $\partial F^*(y')$ and $\partial F^*(y)$.

In our general analysis, we do not set any conditions on $\psi$ and $\phi$, as their role is simply symbolic transfer of dissatisfaction of strong monotonicity into a penalty in our abstract convergence results.

Let us next look at a few examples on how (G-PM) or ($\hbox {F}^*$-PM) might be satisfied. First we have the very well-behaved case of quadratic functions.

#### Example 2.1

$G(x)=\Vert f-Ax\Vert ^2/2$ satisfies (G-PM) with $\Gamma =A^*A$, $\widetilde{\mathcal {K}}=\{0\}$, and $\psi \equiv 0$ for any invertible ${\widetilde{T}}$. Indeed, *G* is differentiable with $\langle \nabla G(x') - \nabla G(x),{\widetilde{T}}^{-1}(x'-x)\rangle =\langle A^*A(x'-x),{\widetilde{T}}^{-1}(x'-x)\rangle =\Vert x'-x\Vert _{{{\widetilde{T}}}^{-1,*}\Gamma }^2$.


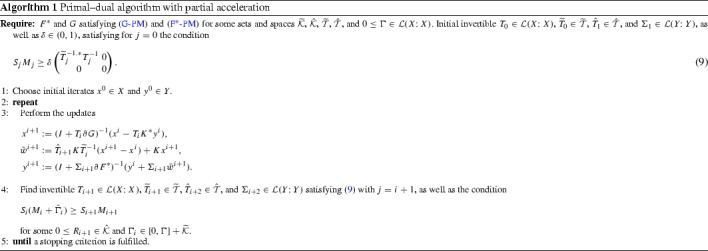


The next lemma demonstrates what can be done when all the parameters are scalar. It naturally extends to functions of the form $G(x_1, x_2)=G(x_1)+G(x_2)$ with corresponding product form parameters.

#### Lemma 2.1

Let $G: X \rightarrow \overline{\mathbb {R}}$ be convex and proper with ${{\mathrm{dom}}}G$ bounded. Then,

8 $$\begin{aligned} G(x')-G(x) \ge \langle \partial G(x),x'-x\rangle + \frac{\gamma }{2}\left( \Vert x'-x\Vert ^2-C_\psi \right) ,\nonumber \\ \end{aligned}$$

for some constant $C_\psi \ge 0$, every $\gamma \ge 0$, and $x, x' \in X$.

#### Proof

We denote $A :={{\mathrm{dom}}}G$. If $x' \not \in A$, we have $G(x')=\infty$, so (8) holds irrespective of $\gamma$ and *C*. If $x \not \in A$, we have $\partial G(x)=\emptyset$, so (8) again holds. We may therefore compute the constants based on $x, x' \in A$. Now, there is a constant *M* such that $\sup _{x \in A} \Vert x\Vert \le M$. Then, $\Vert x'-x\Vert \le 2M$. Thus, if we pick $C=4M^2$, then $(\gamma /2)(\Vert x'-x\Vert ^2-C) \le 0$ for every $\gamma \ge 0$ and $x, x' \in A$. By the convexity of *G*, (8) holds.$\square$

#### Example 2.2

An indicator function $\iota _A$ of a convex bounded set *A* satisfies the conditions of Lemma 2.1. This is generally what we will use and need.

### A General Algorithm and the Idea of Testing

The only change we make to the proximal point formulation (5) of the method (3) is to replace the basic step length or preconditioning operator $M_{\text {basic},i}$ by the operator

10 $$\begin{aligned} M_i :=\begin{pmatrix} T^{-1}_i &{} -K^* \\ -{\hat{T}}_{i+1} K {\widetilde{T}}^{-1}_i &{} \Sigma ^{-1}_{i+1} \end{pmatrix}. \end{aligned}$$

As we have remarked, the operators ${\hat{T}}_{i+1}$ and ${\widetilde{T}}_i$ play the role of $\omega _i$, acting from both sides of *K*. Our proposed algorithm can thus be characterised as solving on each iteration $i \in \mathbb {N}$ for the next iterate $u^{i+1}$ the preconditioned proximal point problem

PP $$\begin{aligned} 0 \in H(u^{i+1}) + M_i(u^{i+1}-u^i). \end{aligned}$$

To study the convergence properties of (PP), we define the *testing operator*

11 $$\begin{aligned} S_i :=\begin{pmatrix} {{\widetilde{T}}}^{-1,*}_i &{}\quad 0 \\ 0 &{}\quad {\hat{T}}_{i+1}^{-1} \end{pmatrix}. \end{aligned}$$

It will turn out that multiplying or ‘testing’ (PP) by this operator will allow us to derive convergence rates. The testing of (PP) by $S_i$ is why we introduced testing into the monotonicity conditions (G-PM) and ($\hbox {F}^*$-PM). If we only tested (PP) with $S_i=I$, we could at most obtain ergodic convergence of the duality gap for the unaccelerated method. But by testing with something appropriate and faster increasing, such as (11), we are able to extract better convergence rates from (PP).

We also set

$$\begin{aligned} \bar{\Gamma }_i= \begin{pmatrix} 2\Gamma _i &{} \quad {\widetilde{T}}_i^*(K{\widetilde{T}}^{-1}_i-{\hat{T}}^{-1}_{i+1}K)^* \\ {\hat{T}}_{i+1} (K{\widetilde{T}}^{-1}_i - {\hat{T}}^{-1}_{i+1} K) &{}\quad 2 R_{i+1} \end{pmatrix}, \end{aligned}$$

for some $\Gamma _i \in [0, \Gamma ]+\widetilde{\mathcal {K}}$ and $R_{i+1} \in \hat{\mathcal {K}}$. We will see in Sect. 2.6 that $\bar{\Gamma }_i$ is a factor of partial strong monotonicity for *H* with respect to testing by $S_i$. With this, taking a fixed $\delta >0$, the properties

C1 $$\begin{aligned} S_i(M_i+\bar{\Gamma }_i)&\ge S_{i+1}M_{i+1}, \quad \text {and} \end{aligned}$$

C2 $$\begin{aligned} S_iM_i&\ge \delta \begin{pmatrix} {{\widetilde{T}}}^{-1,*}_i T^{-1}_i &{} 0 \\ 0 &{} 0 \end{pmatrix} \ge 0, \end{aligned}$$

will turn out to be the crucial defining properties for the convergence rates of the iteration (PP). The method resulting from the combination of (PP), (C1), and (C2) can also be expressed as Algorithm 1. The main steps in developing practical algorithms based on Algorithm 1 will be in the choice of the various step length operators. This will be the content of Sects. 3 and 4. Before this, we expand the conditions (C1) and (C2) to see how they might be satisfied and study abstract convergence results.

### A Simplified Condition

We expand

12 $$\begin{aligned} S_iM_i = \begin{pmatrix} {{\widetilde{T}}}^{-1,*}_i T^{-1}_i &{}\quad - {{\widetilde{T}}}^{-1,*}_i K^* \\ -K{\widetilde{T}}^{-1}_i &{}\quad {\hat{T}}^{-1}_{i+1}\Sigma ^{-1}_{i+1} \end{pmatrix}, \end{aligned}$$

as well as

13 $$\begin{aligned} S_i\bar{\Gamma }_i= \begin{pmatrix} 2 {{\widetilde{T}}}^{-1,*}_i \Gamma _i &{}\quad {{\widetilde{T}}}^{-1,*}_i K^* - K^* {{\hat{T}}}^{-1,*}_{i+1} \\ K{\widetilde{T}}^{-1}_i-{\hat{T}}^{-1}_{i+1}K &{}\quad 2 {\hat{T}}^{-1}_{i+1} R_{i+1} \end{pmatrix}, \end{aligned}$$

and

$$\begin{aligned}&S_i(M_i +\bar{\Gamma }_i)\\&\quad = \begin{pmatrix} {{\widetilde{T}}}^{-1,*}_i(T^{-1}_i +2\Gamma _i) &{}\quad - K^* {{\hat{T}}}^{-1,*}_{i+1} \\ -{\hat{T}}^{-1}_{i+1}K &{}\quad {\hat{T}}^{-1}_{i+1}(\Sigma ^{-1}_{i+1} + 2 R_{i+1}) \end{pmatrix}. \end{aligned}$$

We observe that if $S, T \in \mathcal {L}(X; Y)$, then for arbitrary invertible $Z \in \mathcal {L}(Y; Y)$ a type of Cauchy (or Young) inequality holds, namely

14 $$\begin{aligned} \begin{pmatrix} 0 &{}\quad T^*S \\ S^*T &{}\quad 0 \end{pmatrix}= & {} \begin{pmatrix} 0 &{}\quad T^* Z^* {Z}^{-1,*} S \\ S^* Z^{-1} Z T &{}\quad 0 \end{pmatrix}\nonumber \\\le & {} \begin{pmatrix} T^* Z^* Z T &{}\quad 0 \\ 0 &{}\quad S^* Z^{-1} {Z}^{-1,*} S \end{pmatrix}. \end{aligned}$$

The inequality here can be verified using the basic Cauchy inequality $2\langle x,y\rangle \le \Vert x\Vert ^2 + \Vert y\Vert ^2$. Applying (14) in (12), we see that (C2) is satisfied when

15 $$\begin{aligned}&{\hat{T}}^{-1}_{i+1}\Sigma ^{-1}_{i+1} \ge K Z^{-1}_i {Z}^{-1,*}_i K^*, \quad \text {and}\nonumber \\&\quad (1-\delta ){{\widetilde{T}}}^{-1,*}_i T^{-1}_i \ge {{\widetilde{T}}}^{-1,*}_i Z_i^* Z_i{\widetilde{T}}^{-1}, \end{aligned}$$

for some invertible $Z_i \in \mathcal {L}(X; X)$. The second condition in (15) is satisfied as an equality if

16 $$\begin{aligned} Z_i^*Z_i=(1-\delta )T^{-1}_i {\widetilde{T}}_i. \end{aligned}$$

By the spectral theorem for self-adjoint operators on Hilbert spaces (e.g. [22, Chapter 12]), we can make the choice (16) if

$$\begin{aligned}&T^{-1}_i{\widetilde{T}}_i \in \mathcal {Q} \\&\quad :=\{A \in \mathcal {L}(X; X) \mid A \text { is self-adjoint and positive definite}\}. \end{aligned}$$

Equivalently, by the same spectral theorem, ${\widetilde{T}}^{-1}_iT_i \in \mathcal {Q}$. Therefore, we see from (15) that (C2) holds when 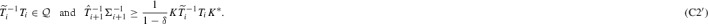 Also, (C1) can be rewritten as 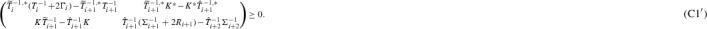


### Basic Convergence Result

Our main result on Algorithm 1 is the following theorem, providing some general convergence estimates. It is, however, important to note that the theorem does not yet directly prove convergence, as its estimates depend on the rate of decrease in $T_N {\widetilde{T}}_N^*$, as well as the rate of increase in the penalty sum $\sum _{i=0}^{N-1} D_{i+1}$ coming from the dissatisfaction of strong convexity. Deriving these rates in special cases will be the topic of Sect. 4.

#### Theorem 2.1

Let us be given $K \in \mathcal {L}(X; Y)$, and convex, proper, lower semicontinuous functionals $G: X \rightarrow \overline{\mathbb {R}}$ and $F^*: Y \rightarrow \overline{\mathbb {R}}$ on Hilbert spaces *X* and *Y*, satisfying (G-PM) and ($\hbox {F}^*$-PM). Pick $\delta \in (0, 1)$, and suppose (C1) and (C2) are satisfied for each $i \in \mathbb {N}$ for some invertible $T_{i} \in \mathcal {L}(X; X)$, ${\widetilde{T}}_{i} \in \widetilde{\mathcal {T}}$, ${\hat{T}}_{i+1} \in \hat{\mathcal {T}}$, and $\Sigma _{i+1} \in \mathcal {L}(Y; Y)$, as well as $\Gamma _i \in [0, \Gamma ] + \widetilde{\mathcal {K}}$ and $R_{i+1} \in \hat{\mathcal {K}}$. Suppose that ${{\widetilde{T}}}^{-1,*}_iT^{-1}_i$ and ${\hat{T}}^{-1}_{i+1}\Sigma ^{-1}_{i+1}$ are self-adjoint. Let ${\widehat{u}}=({\widehat{x}}, {\widehat{y}})$ satisfy (OC). Then, the iterates of Algorithm 1 satisfy

17 $$\begin{aligned} \frac{\delta }{2}\Vert x^N-{\widehat{x}}\Vert _{{{\widetilde{T}}}^{-1,*}_N T^{-1}_N}^2 \le C_0 + \sum _{i=0}^{N-1} {\widetilde{D}}_{i+1}, \quad (N \ge 1), \end{aligned}$$

for

18 $$\begin{aligned}&{\widetilde{D}}_{i+1} :=\psi _{{{\widetilde{T}}}^{-1,*}_i(\Gamma _i-\Gamma )}({x^{i+1}-{\widehat{x}}}) \nonumber \\&\quad +\, \phi _{{\hat{T}}^{-1}_{i+1} R_{i+1}}({y^{i+1}-{\widehat{y}}}), \quad \text {and}\quad C_0 :=\frac{1}{2}\Vert u^0-{\widehat{u}}\Vert _{S_0 M_0}^2.\nonumber \\ \end{aligned}$$

#### Remark 2.1

The term ${\widetilde{D}}_{i+1}$, coming from the dissatisfaction of strong convexity, penalises the basic convergence, which is on the right-hand side of (17) presented by the constant $C_0$. If $T_N {\widetilde{T}}_N$ is of the order $O(1/N^2)$, at least on a subspace, and we can bound the penalty ${\widetilde{D}}_{i+1} \le C$ for some constant *C*, then we clearly obtain mixed $O(1/N^2) + O(1/N)$ convergence rates on the subspace. If we can assume that ${\widetilde{D}}_{i+1}$ actually converges to zero at some rate, then it will even be possible to obtain improved convergence rates. Since typically ${\widetilde{T}}_i, {\hat{T}}_{i+1} \searrow 0$ reduce to scalar factors within ${\widetilde{D}}_{i+1}$, this would require prior knowledge of the rates of convergence $x^i \rightarrow {\widehat{x}}$ and $y^i \rightarrow {\widehat{y}}$. Boundedness of the iterates $\{(x^i, y^i)\}_{i=0}^\infty$, we can, however, usually ensure.

#### Proof

Since $0 \in H({\widehat{u}})$, we have

$$\begin{aligned} \langle H(u^{i+1}),S_i^*(u^{i+1}- {\widehat{u}})\rangle\subset & {} \langle H(u^{i+1})\\&-H({\widehat{u}}),S_i^*(u^{i+1}- {\widehat{u}})\rangle . \end{aligned}$$

Recalling the definition of $S_i$ from (11), and of *H* from (6), it follows

$$\begin{aligned}&\langle H(u^{i+1}),S_i^*(u^{i+1}- {\widehat{u}})\rangle \subset \langle \partial G(x^{i+1})\nonumber \\&\quad -\,\partial G({\widehat{x}}),{\widetilde{T}}^{-1}_i(x^{i+1}- {\widehat{x}})\rangle \\&\quad +\, \langle \partial F^*(y^{i+1})-\partial F^*({\widehat{y}}),{{\hat{T}}_{i+1}}^{-1,*}(y^{i+1}- {\widehat{y}})\rangle \\&\quad +\, \langle K^*(y^{i+1}-{\widehat{y}}),{\widetilde{T}}^{-1}_i(x^{i+1}-{\widehat{x}})\rangle \\&\quad -\, \langle K(x^{i+1}-{\widehat{x}}),{{\hat{T}}_{i+1}}^{-1,*}(y^{i+1}-{\widehat{y}})\rangle . \end{aligned}$$

An application of (G-PM) and ($\hbox {F}^*$-PM) consequently gives

$$\begin{aligned}&\langle H(u^{i+1}),S_i^*(u^{i+1}- {\widehat{u}})\rangle \\&\quad \ge \Vert x^{i+1}-{\widehat{x}}\Vert _{{{\widetilde{T}}}^{-1,*}_i\Gamma _i}^2 +\Vert y^{i+1}-{\widehat{y}}\Vert _{{\hat{T}}^{-1}_{i+i}R_{i+1}}^2\\&\qquad -\,\phi _{{\hat{T}}^{-1}_{i+1}R_{i+1}}({y^{i+1}-{\widehat{y}}}) -\psi _{{{\widetilde{T}}}^{-1,*}_i(\Gamma _i-\Gamma )}({x^{i+1}-{\widehat{x}}})\\&\qquad +\, \langle K {\widetilde{T}}^{-1}_i(x^{i+1}-{\widehat{x}}),y^{i+1}-{\widehat{y}}\rangle \nonumber \\&\qquad -\, \langle {\hat{T}}_{i+1}^{-1}K(x^{i+1}-{\widehat{x}}),y^{i+1}-{\widehat{y}}\rangle . \end{aligned}$$

Using the expression (13) for $S_i\bar{\Gamma }_i$, and (18) for ${\widetilde{D}}_{i+1}$, we thus deduce

19 $$\begin{aligned} \langle H(u^{i+1}),S_i^*(u^{i+1}- {\widehat{u}})\rangle \ge \frac{1}{2}\Vert u^{i+1}-{\widehat{u}}\Vert _{S_i\bar{\Gamma }_i}^2 -{\widetilde{D}}_{i+1}.\nonumber \\ \end{aligned}$$

For arbitrary self-adjoint $M \in \mathcal {L}(X \times Y; X \times Y)$, we calculate

20 $$\begin{aligned}&\langle u^{i+1}-u^i,u^{i+1}-{\widehat{u}}\rangle _M = \frac{1}{2}\Vert u^{i+1}-u^i\Vert _{M}^2\nonumber \\&\quad - \frac{1}{2}\Vert u^i-{\widehat{u}}\Vert _{M}^2 + \frac{1}{2}\Vert u^{i+1}-{\widehat{u}}\Vert _{M}^2. \end{aligned}$$

We observe that $S_iM_i$ in (12) is self-adjoint as we have assumed that ${{\widetilde{T}}}^{-1,*}_iT^{-1}_i$ and ${\hat{T}}^{-1}_{i+1}\Sigma ^{-1}_{i+1}$ are self-adjoint. In consequence, using (20) we obtain

$$\begin{aligned}&\langle M_i(u^i-u^{i+1}),S_i^*(u^{i+1}- {\widehat{u}})\rangle = -\frac{1}{2}\Vert u^{i+1}-u^i\Vert _{S_i M_i}^2\nonumber \\&\quad + \frac{1}{2}\Vert u^i-{\widehat{u}}\Vert _{S_i M_i}^2 - \frac{1}{2}\Vert u^{i+1}-{\widehat{u}}\Vert _{S_i M_i}^2. \end{aligned}$$

Using (C1) to estimate $\frac{1}{2}\Vert u^{i+1}-{\widehat{u}}\Vert _{S_i M_i}^2$ and (C2) to eliminate $\frac{1}{2}\Vert u^{i+1}-u^i\Vert _{S_i M_i}^2$ yields

21 $$\begin{aligned}&\langle M_i(u^i-u^{i+1}),S_i^*(u^{i+1}- {\widehat{u}})\rangle \le \frac{1}{2}\Vert u^i-{\widehat{u}}\Vert _{S_i M_i}^2\nonumber \\&\quad - \frac{1}{2}\Vert u^{i+1}-{\widehat{u}}\Vert _{S_{i+1} M_{i+1}}^2 + \frac{1}{2}\Vert u^{i+1}-{\widehat{u}}\Vert _{S_i\bar{\Gamma }_i}^2. \end{aligned}$$

Combining (19) and (21) through (PP), we thus obtain

22 $$\begin{aligned} \frac{1}{2}\Vert u^{i+1}-{\widehat{u}}\Vert _{S_{i+1} M_{i+1}}^2 \le \frac{1}{2}\Vert u^i-{\widehat{u}}\Vert _{S_i M_i}^2 +{\widetilde{D}}_{i+1}. \end{aligned}$$

Summing (22) over $i=1,\ldots ,N-1$, and applying (C2) to estimate $S_N M_N$ from below, we obtain (17).$\square$

## Scalar Off-diagonal Updates and the Ergodic Duality Gap

One relatively easy way to satisfy (G-PM), ($\hbox {F}^*$-PM), (C1) and (C2) is to take the ‘off-diagonal’ step length operators ${\hat{T}}_i$ and ${\widetilde{T}}_i$ as equal scalars. Another good starting point would be to choose ${\widetilde{T}}_i=T_i$. We, however, do not explore this route in the present work. Instead, we now specialise Theorem 2.1 to the scalar case. We then explore ways to add estimates of the ergodic duality gap into (17). While this would be possible in the general framework through convexity notions analogous to (G-PM) and ($\hbox {F}^*$-PM), the resulting gap would not be particularly meaningful. We therefore concentrate on the scalar off-diagonal updates to derive estimates on the ergodic duality gap.
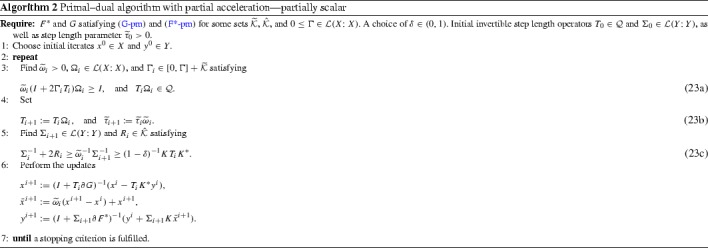


### Scalar Specialisation of Algorithm 1

We take both ${\widetilde{T}}_i={\widetilde{\tau }}_i I$, and ${\hat{T}}_i={\widetilde{\tau }}_i I$ for some ${\widetilde{\tau }}_i > 0$. With

$$\begin{aligned} {\widetilde{\omega }}_i :={\widetilde{\tau }}_{i+1}/{\widetilde{\tau }}_i, \end{aligned}$$

the condition ($\hbox {C2}'$) then becomes 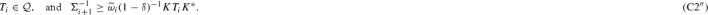


The off-diagonal terms cancelling out ($\hbox {C1}'$) on the other hand become 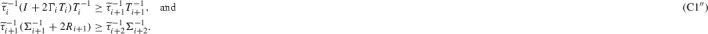 Observe also that $M_i$ is under this setup self-adjoint if $T_i$ and $\Sigma _{i+1}$ are.

For simplicity, we now assume $\phi$ and $\psi$ to satisfy the identities

24 $$\begin{aligned} \psi _T(-x)= & {} \psi _T(x), \quad \text {and}\nonumber \\ \psi _{\alpha T}(x)= & {} \alpha \psi _T(x), \quad (x \in X;\, 0 < \alpha \in \mathbb {R}). \end{aligned}$$

The monotonicity conditions (G-PM) and ($\hbox {F}^*$-PM) then simplify into

G-pm $$\begin{aligned} \langle \partial G(x') - \partial G(x),x'-x\rangle \ge \Vert x'-x\Vert _{\Gamma '}^2-\psi _{\Gamma '-\Gamma }({x'-x}), \end{aligned}$$

holding for all $x, x' \in X$, and $\Gamma ' \in [0, \Gamma ] + \widetilde{\mathcal {K}}$; and

F*-pm $$\begin{aligned} \langle \partial F^*(y') - \partial F^*(y) ,y'-y\rangle \ge \Vert y'-y\Vert _{R}^2-\phi _{R}({y'-y}), \end{aligned}$$

holding for all $y, y' \in Y$, and $R \in \hat{\mathcal {K}}$.

We have thus converted the main conditions (C2), (C1), (G-PM), and ($\hbox {F}^*$-PM) of Theorem 2.1 into the respective conditions ($\hbox {C2}''$), ($\hbox {C1}''$), (G-pm), and ($\hbox {F}^*-\hbox {pm}$). Rewriting ($\hbox {C1}''$) in terms of $0 < \Omega _i \in \mathcal {L}(X; X)$ and ${\widetilde{\omega }}_i > 0$ satisfying

$$\begin{aligned} T_{i+1}=T_i\Omega _i \quad \text {and}\quad {\widetilde{\tau }}_{i+1}={\widetilde{\tau }}_i{\widetilde{\omega }}_i, \end{aligned}$$

we reorganise ($\hbox {C1}''$) and ($\hbox {C2}''$) into the parameter update rules (23) of Algorithm 2. For ease of expression, we introduce there $\Sigma _0$ and $R_0$ as dummy variables that are not used anywhere else. Equating $\bar{w}^{i+1}=K\bar{x}^{i+1}$, we observe that Algorithm 2 is an instance of Algorithm 1.

#### Example 3.1

(The method of Chambolle and Pock) Let *G* be strongly convex with factor $\gamma \ge 0$. We take $T_i=\tau _i I$, ${\widetilde{T}}_i=\tau _i I$, ${\hat{T}}_i=\tau _i I$, and $\Sigma _{i+1}=\sigma _{i+1} I$ for some scalars $\tau _i, \sigma _{i+1}>0$. The conditions (G-pm) and ($\hbox {F}^*-\hbox {pm}$) then hold with $\psi \equiv 0$ and $\phi \equiv 0$, while ($\hbox {C2}''$) and ($\hbox {C1}''$) reduce with $R_{i+1}=0$, $\Gamma _i=\gamma I$, $\Omega _i=\omega _i I$, and ${\widetilde{\omega }}_i=\omega _i$ into

$$\begin{aligned}&\omega _i^2(1+2\gamma \tau _i) \ge 1, \quad \text {and}\\&(1-\delta )/\Vert K\Vert ^2 \ge \tau _{i+2}\sigma _{i+2} \ge \tau _{i+1}\sigma _{i+1}. \end{aligned}$$

Updating $\sigma _{i+1}$ such that the last inequality holds as an equality, we recover the accelerated PDHGM (3) $+$ (4). If $\gamma =0$, we recover the unaccelerated PDHGM.

### The Ergodic Duality Gap and Convergence

To study the convergence of an ergodic duality gap, we now introduce convexity notions analogous to (G-pm) and ($\hbox {F}^*-\hbox {pm}$). Namely, we assume

G-pc $$\begin{aligned} G(x')-G(x)&\ge \langle \partial G(x),x'-x\rangle + \frac{1}{2}\Vert x'-x\Vert _{\Gamma '}^2\\&\quad -\frac{1}{2}\psi _{\Gamma '-\Gamma }({x'-x}), \end{aligned}$$

to hold for all $x, x' \in X$ and $\Gamma ' \in [0, \Gamma ]+\widetilde{\mathcal {K}}$ and 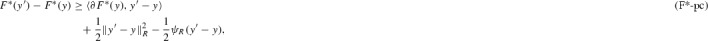 to hold for all $y, y' \in Y$ and $R \in \hat{\mathcal {K}}$. Clearly these imply (G-pm) and ($\hbox {F}^*-\hbox {pm}$).

To define an ergodic duality gap, we set

25 $$\begin{aligned} {\widetilde{q}}_N :=\sum _{i=0}^{N-1} {\widetilde{\tau }}_i^{-1}, \quad \text {and}\quad {\hat{q}}_N :=\sum _{i=0}^{N-1} {\widetilde{\tau }}_{i+1}^{-1}, \end{aligned}$$

and define the weighted averages

$$\begin{aligned} x_N :=\widetilde{q}_N^{-1} \sum _{i=0}^{N-1} {\widetilde{\tau }}_i^{-1} x^{i+1}, \quad \text {and}\quad y_N :={\hat{q}}_N^{-1} \sum _{i=0}^{N-1} {\widetilde{\tau }}_{i+1}^{-1} y^{i+1}. \end{aligned}$$

With these, the ergodic duality gap at iteration *N* is defined as the duality gap for $(x_N, Y_N)$, namely

$$\begin{aligned} \mathcal {G}^{N}:= & {} \bigl (G(x_N) + \langle {\widehat{y}},Kx_N\rangle - F^*({\widehat{y}})\bigr )\nonumber \\&-\bigl (G({\widehat{x}}) + \langle y_N,K {\widehat{x}}\rangle - F^*(y_N)\bigr ), \end{aligned}$$

and we have the following convergence result.

#### Theorem 3.1

Let us be given $K \in \mathcal {L}(X; Y)$, and convex, proper, lower semicontinuous functionals $G: X \rightarrow \overline{\mathbb {R}}$ and $F^*: Y \rightarrow \overline{\mathbb {R}}$ on Hilbert spaces *X* and *Y*, satisfying (G-pc) and ($\hbox {F}^*\hbox {-pc}$) for some sets $\widetilde{\mathcal {K}}$, $\hat{\mathcal {K}}$, and $0 \le \Gamma \in \mathcal {L}(X; X)$. Pick $\delta \in (0, 1)$, and suppose ($\hbox {C2}''$) and ($\hbox {C1}''$) are satisfied for each $i \in \mathbb {N}$ for some invertible self-adjoint $T_i \in \mathcal {Q}$, $\Sigma _{i} \in \mathcal {L}(Y; Y)$, 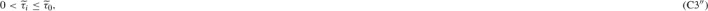 as well as $\Gamma _i \in \lambda ([0, \Gamma ] + \widetilde{\mathcal {K}})$ and $R_{i} \in \lambda \hat{\mathcal {K}}$ for $\lambda =1/2$. Let ${\widehat{u}}=({\widehat{x}}, {\widehat{y}})$ satisfy (OC). Then, the iterates of Algorithm 2 satisfy

26 $$\begin{aligned} \frac{\delta }{2}\Vert x^N-{\widehat{x}}\Vert _{{\widetilde{\tau }}_N^{-1} T^{-1}_N}^2 + {\widetilde{q}}_N\mathcal {G}^{N} \le C_0 + \sum _{i=0}^{N-1} D_{i+1}. \end{aligned}$$

Here $C_0$ is as in (18), and

27 $$\begin{aligned} D_{i+1} :={\widetilde{\tau }}_i^{-1}\psi _{\Gamma _i-\lambda \Gamma }({x^{i+1}-{\widehat{x}}}) + {\widetilde{\tau }}_{i+1}^{-1}\phi _{R_{i+1}}({y^{i+1}-{\widehat{y}}}).\nonumber \\ \end{aligned}$$

If only (G-pm) and ($\hbox {F}^*-\hbox {pm}$) hold instead of (G-pc) and ($\hbox {F}^*-\hbox {pc}$), and we take $\lambda =1$, then (26) holds with the modification $\mathcal {G}^{N} :=0$.

#### Remark 3.1

For convergence of the gap, we must accelerate less (factor 1 / 2 on $\Gamma _i$).

#### Example 3.2

(*No acceleration*) Consider Example 3.1, where $\psi \equiv 0$ and $\phi \equiv 0$. If $\gamma =0$, we get ergodic convergence of the duality gap at rate *O*(1 / *N*). Indeed, we are in the scalar step setting, with ${\widetilde{\tau }}_j={\widetilde{\tau }}_j=\tau _0$. Thus, presently ${\widetilde{q}}_N=N\tau _0$.

#### Example 3.3

(*Full acceleration*) With $\gamma >0$ in Example 3.1, we know from [3, Corollary 1] that

28 $$\begin{aligned} \lim _{N \rightarrow \infty } N\tau _N\gamma =1. \end{aligned}$$

Thus, ${\widetilde{q}}_N$ is of the order $\Omega (N^2)$, while ${\widetilde{\tau }}_N T_N = \tau _N^2 I$ is of the order $O(1/N^2)$. Therefore, (26) shows $O(1/N^2)$ convergence of the squared distance to solution. For $O(1/N^2)$ convergence of the ergodic duality gap, we need to slow down (4) to $\omega _i = 1/\sqrt{1+\gamma \tau _i}$.

#### Remark 3.2

The result (28) can be improved to estimate $\tau _N \le C_\tau /N$ without a qualifier $N \ge N_0$. Indeed, from [3, Lemma 2] we know the following for the rule $\omega _i=1/\sqrt{1+2\gamma \tau _i}$: given $\lambda >0$ and $N \ge 0$ with $\gamma \tau _N \le \lambda$, for any $\ell \ge 0$ holds

$$\begin{aligned} \frac{1}{\gamma \tau _N} + \frac{\ell }{1+\lambda } \le \frac{1}{\gamma \tau _{N+\ell }} \le \frac{1}{\gamma \tau _N} + \ell . \end{aligned}$$

If we pick $N=0$ and $\lambda =\gamma \tau _0$, this says

$$\begin{aligned} \frac{1}{\gamma \tau _0} + \frac{\ell }{1+\gamma \tau _0} \le \frac{1}{\gamma \tau _{\ell }} \le \frac{1}{\gamma \tau _0} + \ell . \end{aligned}$$

The first inequality gives $\tau _\ell \le (1+\gamma \tau _0)/(\tau ^{-1}_0+\gamma \ell )\le (\gamma ^{-1}+\tau _0)/\ell$.

Therefore, $\tau _N \le C_\tau /N$ for $C_\tau :=\gamma ^{-1}+\tau _0$. Moreover, the second inequality gives $\tau ^{-1}_N \le \tau ^{-1}_0 + \gamma N$.

#### Proof

(*Theorem* 3.1) The non-gap estimate in the last paragraph of the theorem statement, where $\lambda =1$, we modify $\mathcal {G}_N :=0$, is a direct consequence of Theorem 2.1. We therefore concentrate on the estimate that includes the gap, and fix $\lambda = 1/2$. We begin by expanding

$$\begin{aligned}&\langle H(u^{i+1}),S_i^*(u^{i+1}- {\widehat{u}})\rangle \\&\quad = {\widetilde{\tau }}_i^{-1} \langle \partial G(x^{i+1}),x^{i+1}- {\widehat{x}}\rangle + {\widetilde{\tau }}_{i+1}^{-1} \langle \partial F^*(y^{i+1}),y^{i+1}- {\widehat{y}}\rangle \\&\qquad +\, {\widetilde{\tau }}_i^{-1}\langle K^*y^{i+1},x^{i+1}-{\widehat{x}}\rangle - {\widetilde{\tau }}_{i+1}^{-1} \langle K x^{i+1},y^{i+1}-{\widehat{y}}\rangle \end{aligned}$$

Since then $\Gamma _i \in ([0, \Gamma ] + \widetilde{\mathcal {K}})/2$, and $R_{i+1} \in \hat{\mathcal {K}}/2$, we may take $\Gamma '=2\Gamma _i$ and $R=2R_{i+1}$ in (G-pc) and $\hbox {F}^*-\hbox {pc}$. It follows

$$\begin{aligned}&\langle H(u^{i+1}),S_i^*(u^{i+1}- {\widehat{u}})\rangle \\&\quad \ge {\widetilde{\tau }}_i^{-1} \bigl ( G(x^{i+1})- G({\widehat{x}})\\&\qquad +\, \frac{1}{2}\Vert x^{i+1}-{\widehat{x}}\Vert _{2\Gamma _i}^2 - \frac{1}{2}\psi _{2\Gamma _i-\Gamma }({x^{i+1}-{\widehat{x}}}) \bigr )\\&\qquad +\, {\widetilde{\tau }}_{i+1}^{-1} \bigl ( F^*(y^{i+1})- F^*({\widehat{y}}) + \frac{1}{2}\Vert y^{i+1}-{\widehat{y}}\Vert _{2R_{i+1}}^2\\&\qquad -\, \frac{1}{2}\phi _{2R_{i+1}}({y^{i+1}-{\widehat{y}}}) \bigr ) - {\widetilde{\tau }}_i^{-1}\langle y^{i+1},K{\widehat{x}}\rangle \\&\qquad +\, {\widetilde{\tau }}_{i+1}^{-1}\langle {\widehat{y}},K x^{i+1}\rangle + ({\widetilde{\tau }}_i^{-1} - {\widetilde{\tau }}_{i+1}^{-1})\langle y^{i+1},K x^{i+1}\rangle . \end{aligned}$$

Using (2) and (24), we can make all of the factors ‘2’ and ‘1/2’ in this expression annihilate each other. With $D_{i+1}$ as in (27) and $\lambda =1/2$, we therefore have

$$\begin{aligned}&\langle H(u^{i+1}),S_i^*(u^{i+1}- {\widehat{u}})\rangle \\&\quad \ge {\widetilde{\tau }}_i^{-1}\left( G(x^{i+1})-G({\widehat{x}})+\langle {\widehat{y}},Kx^{i+1}\rangle \right) \\&\qquad +\, \Vert x^{i+1}-{\widehat{x}}\Vert _{{\widetilde{\tau }}_i^{-1} \Gamma _i}^2\\&\qquad +\, {\widetilde{\tau }}_{i+1}^{-1}\left( F^*(y^{i+1})-F^*({\widehat{y}})-\langle y^{i+1},K{\widehat{x}}\rangle \right) \\&\qquad +\, \Vert y^{i+1}-{\widehat{y}}\Vert _{{\widetilde{\tau }}_{i+1}^{-1}R_{i+1}}^2\\&\qquad +\, ({\widetilde{\tau }}_i^{-1} - {\widetilde{\tau }}_{i+1}^{-1})\left( \langle y^{i+1}-{\widehat{y}},K(x^{i+1}-{\widehat{x}})\rangle \right. \\&\qquad \left. -\, \langle {\widehat{y}},K{\widehat{x}}\rangle \right) - D_{i+1}. \end{aligned}$$

A little bit of reorganisation and referral to (13) for the expansion of $S_i\bar{\Gamma }_i$ thus gives

29 $$\begin{aligned}&\langle H(u^{i+1}),S_i^*(u^{i+1}- {\widehat{u}})\rangle \nonumber \\&\quad \ge {\widetilde{\tau }}_i^{-1} \left( G(x^{i+1})-G({\widehat{x}}) + \langle {\widehat{y}},Kx^{i+1}\rangle \right) \nonumber \\&\qquad +\, {\widetilde{\tau }}_{i+1}^{-1}\left( F^*(y^{i+1})-F^*({\widehat{y}}) - \langle y^{i+1},K{\widehat{x}}\rangle \right) \nonumber \\&\qquad -\, ({\widetilde{\tau }}_i^{-1} - {\widetilde{\tau }}_{i+1}^{-1})\langle {\widehat{y}},K{\widehat{x}}\rangle + \frac{1}{2}\Vert u^{i+1}-{\widehat{u}}\Vert _{S_i \bar{\Gamma }_i}^2 - D_{i+1}.\nonumber \\ \end{aligned}$$

Let us write

$$\begin{aligned}&\mathcal {G}^i_+(u^{i+1}, {\widehat{u}}) :=\bigl ({\widetilde{\tau }}_i^{-1}G(x^{i+1})+ {\widetilde{\tau }}_i^{-1}\langle {\widehat{y}},Kx^{i+1}\rangle -{\widetilde{\tau }}_{i}^{-1}F^*({\widehat{y}})\bigr )\\&\quad -\bigl ({\widetilde{\tau }}_{i+1}^{-1}G({\widehat{x}}) + {\widetilde{\tau }}_{i+1}^{-1}\langle y^{i+1},K{\widehat{x}}\rangle - {\widetilde{\tau }}_{i+1}^{-1}F^*(y^{i+1})\bigr ). \end{aligned}$$

Observing here the switches between the indices ${i+1}$ and *i* of the step length parameters in comparison with the last step of (29), we thus obtain

30 $$\begin{aligned} \langle H(u^{i+1}),S_i(u^{i+1}- {\widehat{u}})\rangle\ge & {} \mathcal {G}^i_+(u^{i+1}, {\widehat{u}}) - \mathcal {G}^i_+({\widehat{u}},{\widehat{u}})\nonumber \\&+\, \frac{1}{2}\Vert u^{i+1}-{\widehat{u}}\Vert _{S_i\bar{\Gamma }_i}^2 - D_{i+1}.\nonumber \\ \end{aligned}$$

We note that $S_iM_i$ in (12) is self-adjoint as we have assumed $T_i$ and $\Sigma _{i+1}$ to be, and taken ${\widetilde{T}}_i$ and ${\hat{T}}_{i+1}$ to be scalars times the identity. We therefore deduce from the proof of Theorem 2.1 that (21) holds. Using (PP) to combine (21) and (30), we thus deduce

$$\begin{aligned}&\frac{1}{2}\Vert u^{i+1}- {\widehat{u}}\Vert _{S_{i+1} M_{i+1}}^2 + \mathcal {G}^i_+(u^{i+1}, {\widehat{u}})\nonumber \\&\quad - \mathcal {G}^i_+({\widehat{u}}, {\widehat{u}}) \le \frac{1}{2}\Vert u^i- {\widehat{u}}\Vert _{S_{i} M_{i}}^2 + D_{i+1}. \end{aligned}$$

Summing this for $i=0,\ldots ,N-1$ gives with $C_0$ from (27) the estimate

31 $$\begin{aligned}&\frac{1}{2}\Vert u^N-{\widehat{u}}\Vert _{S_{N} M_{N}}^2 + \sum _{i=0}^{N-1} \left( \mathcal {G}^i_+(u^{i+1}, {\widehat{u}}) -\mathcal {G}^i_+({\widehat{u}}, {\widehat{u}})\right) \nonumber \\&\quad \le C_0 +\sum _{i=0}^{N-1} D_{i+1}. \end{aligned}$$

We want to estimate the sum of the gaps $\mathcal {G}^i_+$ in (31). Using the convexity of *G* and $F^*$, we observe

32 $$\begin{aligned}&\sum _{i=0}^{N-1} {\widetilde{\tau }}_{i}^{-1} G(x^{i+1}) \ge {\widetilde{q}_N} G(x_N), \quad \text {and}\nonumber \\&\sum _{i=0}^{N-1} {\widetilde{\tau }}_{i+1}^{-1} F^*(y^{i+1}) \ge {{\hat{q}}_N} F^*(y_N). \end{aligned}$$

Also, by (25) and simple reorganisation

33 $$\begin{aligned} \sum _{i=0}^{N-1} {\widetilde{\tau }}_{i+1}^{-1}G({\widehat{x}})&= {\widetilde{q}_N} G({\widehat{x}}) + {\widetilde{\tau }}_{N}^{-1} G({\widehat{x}}) - {\widetilde{\tau }}_0^{-1} G({\widehat{x}}), \quad \text {and} \end{aligned}$$

34 $$\begin{aligned} \sum _{i=0}^{N-1} {\widetilde{\tau }}_{i}^{-1} F^*({\widehat{y}})&= {{\hat{q}}_N} F^*(y_N) -{\widetilde{\tau }}_N^{-1} F^*({\widehat{y}}) +{\widetilde{\tau }}_0^{-1} F^*({\widehat{y}}). \end{aligned}$$

All of (32)–(34) together give

$$\begin{aligned}&\sum _{i=0}^{N-1} \mathcal {G}^i_+(u^{i+1},{\widehat{u}})\\&\quad \ge \bigr ({\widetilde{q}_N}G(x_N) + {\widetilde{q}}_N\langle {\widehat{y}},Kx_N\rangle - {{\hat{q}}_N}F^*({\widehat{y}}) \bigl )\\&\qquad -\, \bigl ({\widetilde{q}_N}G({\widehat{x}}) + {\hat{q}}_N\langle y_N,K{\widehat{x}}\rangle - {\hat{q}}_NF^*(y_N)\bigr )\\&\qquad + \left( {\widetilde{\tau }}_N^{-1}G({\widehat{x}}) - {\widetilde{\tau }}_0^{-1}G({\widehat{x}}) + {\widetilde{\tau }}_N^{-1}F^*_{{{\hat{T}}}^{-1,*}_{N}}({\widehat{x}}) - {\widetilde{\tau }}_0^{-1}F^*({\widehat{y}}) \right) . \end{aligned}$$

Another use of (25) gives

$$\begin{aligned}&\sum _{i=0}^{N-1} \mathcal {G}^i_+({\widehat{u}},{\widehat{u}})\\&\quad = ({\widetilde{q}}_N - {\hat{q}}_N) \langle {\widehat{y}},K{\widehat{x}}\rangle \nonumber \\&\qquad + \left( {\widetilde{\tau }}_N^{-1} G({\widehat{x}}) - \widetilde{\tau }_0^{-1} G({\widehat{x}}) + {\widetilde{\tau }}_N^{-1} F^*({\widehat{x}}) - \widetilde{\tau }_0^{-1} F^*({\widehat{y}}) \right) . \end{aligned}$$

Thus,

35 $$\begin{aligned} \sum _{i=0}^{N-1} \bigl (\mathcal {G}^i_+(u^{i+1},{\widehat{u}})-\mathcal {G}^i_+({\widehat{u}},{\widehat{u}})\bigr ) \ge {\widetilde{q}}_N \mathcal {G}^{N} + r_N, \end{aligned}$$

where the remainder

$$\begin{aligned}\nonumber r_N =({\widetilde{q}}_N-{\hat{q}}_N)\left( F^*({\hat{y}})-F^*(y_N) - \langle {\hat{y}}-y_N,K {\hat{x}}\rangle \right) . \end{aligned}$$

At a solution ${\widehat{u}}=({\widehat{x}}, {\widehat{y}})$ to (OC), we have $K {\hat{x}} \in \partial F^*({\hat{y}})$, so $r_N \ge 0$ provided ${\widetilde{q}}_N \le {\hat{q}}_N$. But ${\widetilde{q}}_N-{\hat{q}}_N=\widetilde{\tau }^{-1}_0 - \widetilde{\tau }^{-1}_{N}$, so this is guaranteed by our assumption ($\hbox {C3}''$). Using (35) in (31) therefore gives

36 $$\begin{aligned} \frac{1}{2}\Vert u^N-{\widehat{u}}\Vert _{S_{N} M_{N}}^2 + {\widetilde{q}}_N \mathcal {G}^{N} + r_N \le C_0 + \sum _{i=0}^{N-1} D_{i+1}. \end{aligned}$$

A referral to (C2) to estimate $S_NM_N$ from below shows (26), concluding the proof. $\square$

## Convergence Rates in Special Cases

To derive a practical algorithm, we need to satisfy the update rules (C1) and (C2), as well as the partial monotonicity conditions (G-PM) and ($\hbox {F}^*$-PM). As we have already discussed in Sect. 3, this can be done when for some ${\widetilde{\tau }}_i>0$ we set

37 $$\begin{aligned} {\widetilde{T}}_i={\widetilde{\tau }}_i I, \quad \text {and}\quad {\hat{T}}_i={\widetilde{\tau }}_i I. \end{aligned}$$

The result of these choices is Algorithm 2, whose convergence we studied in Theorem 3.1. Our task now is to verify its conditions, in particular (G-pc) and $\hbox {F}^*-\hbox {pc}$ [alternatively ($\hbox {F}^*-\hbox {pm}$) and (G-pm)], as well as ($\hbox {C1}''$), ($\hbox {C2}''$), and ($\hbox {C3}''$) for $\Gamma$ of the projection form $\gamma P$.

### An Approach to Updating $\Sigma$

We have not yet defined an explicit update rule for $\Sigma _{i+1}$, merely requiring that it has to satisfy ($\hbox {C2}''$) and ($\hbox {C1}''$). The former in particular requires

$$\begin{aligned} \Sigma ^{-1}_{i+1} \ge {\widetilde{\omega }}_i (1-\delta )^{-1} K T_i K^*. \end{aligned}$$

Hiring the help of some linear operator $\mathcal {F} \in \mathcal {L}(\mathcal {L}(Y; Y)$; $\mathcal {L}(Y;Y))$ satisfying

38 $$\begin{aligned} \mathcal {F}(K T_i K^*) \ge K T_i K^*, \end{aligned}$$

our approach is to define

39 $$\begin{aligned} \Sigma ^{-1}_{i+1} :={{\widetilde{\omega }}_i}(1-\delta )^{-1} \mathcal {F}(K T_i K^*). \end{aligned}$$

Then, ($\hbox {C2}''$) is satisfied provided $T^{-1}_i \in \mathcal {Q}$. Since ${\widetilde{\tau }}_{i+1}^{-1}\Sigma _{i+1}^{-1} ={\widetilde{\tau }}_i^{-1}(1-\delta )^{-1} \mathcal {F}(K T_i K^*),$ the condition ($\hbox {C1}''$) reduces into the satisfaction for each $i \in \mathbb {N}$ of

40a $$\begin{aligned}&{\widetilde{\tau }}_i^{-1} (I + 2 \Gamma T_i) T^{-1}_i - {\widetilde{\tau }}_{i+1}^{-1} T^{-1}_{i+1} \ge -2{\widetilde{\tau }}_i^{-1}(\Gamma _i-\Gamma ), \quad \text {and} \end{aligned}$$

40b $$\begin{aligned}&\frac{1}{1-\delta }\left( {\widetilde{\tau }}_{i}^{-1}\mathcal {F}\left( KT_{i}K^*\right) - {\widetilde{\tau }}_{i+1}^{-1}\mathcal {F}\left( KT_{i+1} K^*\right) \right) \nonumber \\&\quad \ge -2 {\widetilde{\tau }}_{i+1}^{-1} R_{i+1}. \end{aligned}$$

To apply Theorem 3.1, all that remains is to verify in special cases the conditions (40) together with ($\hbox {C3}''$) and the partial strong convexity conditions (G-pc) and $\hbox {F}^*-\hbox {pc}$.

### When $\Gamma$ is a Multiple of a Projection

We now take $\Gamma =\bar{\gamma }P$ for some $\bar{\gamma }>0$, and a projection operator $P \in \mathcal {L}(X; X)$: idempotent, $P^2=P$, and self-adjoint, $P^*=P$. We let $P^\perp :=I-P$. Then, $P^\perp P=P P^\perp =0$. With this, we assume that $\widetilde{\mathcal {K}}$ is such that for some $\bar{\gamma }^\perp >0$ holds

41 $$\begin{aligned}{}[0, \bar{\gamma }^\perp P^\perp ] \subset \widetilde{\mathcal {K}}. \end{aligned}$$

To unify our analysis for gap and non-gap estimates of Theorem 3.1, we now pick $\lambda =1/2$ in the former case, and $\lambda =1$ in the latter. We then pick $0 \le \gamma \le \lambda \bar{\gamma }$, and $0 \le \gamma _i^\perp \le \lambda \bar{\gamma }^\perp$, and set

42 $$\begin{aligned} T_i= & {} \tau _i P+\tau _i^\perp P^\perp , \quad \Omega _i=\omega _i P+\omega _i^\perp P^\perp , \quad \text {and}\nonumber \\ \Gamma _i= & {} \gamma P + \gamma _i^\perp P^\perp . \end{aligned}$$

With this, $\tau _i, \tau _i^\perp > 0$ guarantee $T_i \in {\mathcal {Q}}$. Moreover, $T_i$ is self-adjoint. Moreover, $\Gamma _i \in \lambda ([0, \Gamma ] + \widetilde{\mathcal {K}})$, exactly as required in both the gap and the non-gap cases of Theorem 3.1.

Since

$$\begin{aligned} KT_{i}K^*= & {} \tau _{i} KPK^* +\tau _{i}^\perp KP^\perp K^*\\= & {} (\tau _{i} - \tau _{i}^\perp ) KPK^* + \tau _{i}^\perp KK^*, \end{aligned}$$

we are encouraged to take

43 $$\begin{aligned} {\mathcal {F}}(K T_{i} K^*) :=\max \{0, \tau _{i} - \tau _{i}^\perp \} \Vert KP\Vert ^2 I + \tau _{i}^\perp \Vert K\Vert ^2 I.\nonumber \\ \end{aligned}$$

Observe that (43) satisfies (38). Inserting (43) into (39), we obtain

44 $$\begin{aligned} \Sigma _{i+1}= & {} \sigma _{i+1} I \quad \text {with}\nonumber \\ \sigma ^{-1}_{i+1}= & {} \frac{{\widetilde{\omega }}_i}{1-\delta }\left( \max \{0, \tau _{i} - \tau _{i}^\perp \} \Vert KP\Vert ^2 + \tau _i^\perp \Vert K\Vert ^2\right) .\nonumber \\ \end{aligned}$$

Since $\Sigma _{i+1}$ is now equivalent to a scalar, (40b), we also take $R_{i+1}=\rho _{i+1} I$, assuming for some $\bar{\rho }>0$ that

$$\begin{aligned}{}[0, \bar{\rho }I] \subset \hat{\mathcal {K}}. \end{aligned}$$

Setting

$$\begin{aligned} \eta _i :={\widetilde{\tau }}_i^{-1}\max \{0, \tau _{i} - \tau _{i}^\perp \} - {\widetilde{\tau }}_{i+1}^{-1}\max \{0, \tau _{i+1} - \tau _{i+1}^\perp \} \end{aligned}$$

we thus expand (40) as

45a $$\begin{aligned}&{\widetilde{\tau }}_i^{-1} (1 + 2 \gamma \tau _i) \tau ^{-1}_i - {\widetilde{\tau }}_{i+1} \tau ^{-1}_{i+1} \ge 0, \end{aligned}$$

45b $$\begin{aligned}&{\widetilde{\tau }}_i^{-1}\tau _i^{\perp ,-1} - {\widetilde{\tau }}_{i+1}^{-1} \tau _{i+1}^{\perp ,-1} \ge -2 {\widetilde{\tau }}_i^{-1} \gamma _i^\perp , \end{aligned}$$

45c $$\begin{aligned}&\frac{1}{1-\delta } \left( \eta _i \Vert KP\Vert ^2+({\widetilde{\tau }}_i^{-1} \tau _i^\perp -{\widetilde{\tau }}_{i+1}^{-1} \tau _{i+1}^\perp )\Vert K\Vert ^2\right) \nonumber \\&\quad \ge -2 {\widetilde{\tau }}_{i+1}^{-1} \rho _{i+1}. \end{aligned}$$

We are almost ready to state a general convergence result for projective $\Gamma$. However, we want to make one more thing more explicit. Since the choices (42) satisfy

$$\begin{aligned}&\Gamma _i-\lambda \Gamma =(\gamma -\lambda \bar{\gamma })P + \gamma _i^\perp P^\perp \le \gamma _i^\perp P^\perp \\&\quad \text {and}\quad R_{i+1}=\rho _{i+1} I, \end{aligned}$$

we suppose for simplicity that

46 $$\begin{aligned} \psi _{\Gamma _i-\lambda \Gamma }(x) =\gamma _i^\perp \psi ^\perp (P^\perp x) \quad \text {and}\quad \phi _{R_{i+1}}(y)=\rho _{i+1} \phi (y)\nonumber \\ \end{aligned}$$

for some $\psi ^\perp : P^\perp X \rightarrow \mathbb {R}$ and $\phi : Y \rightarrow \mathbb {R}$. The conditions (G-pc) and $\hbox {F}^*-\hbox {pc}$ reduce in this case to the satisfaction for some $\bar{\gamma }, \bar{\gamma }^\perp , \bar{\rho }>0$ of
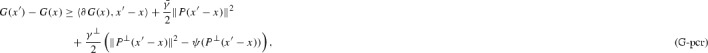
for all $x, x' \in X$ and $0 \le \gamma ^\perp \le \bar{\gamma }^\perp$, as well as of 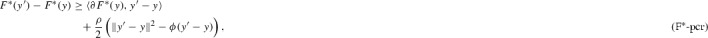 for all $y, y' \in Y$ and $0 \le \rho \le \bar{\rho }$. Analogues of (G-pm) and ($\hbox {F}^*-\hbox {pm}$) can be formed.

To summarise the findings of this section, we state the following proposition.

#### Proposition 4.1

Suppose (G-pcr) and ($\hbox {F}^*$-pcr) hold for some projection operator $P \in \mathcal {L}(X; X)$ and scalars $\bar{\gamma }, \bar{\gamma }^\perp , \bar{\rho }> 0$. With $\lambda =1/2$, pick $\gamma \in [0, \lambda \bar{\gamma }]$. For each $i \in \mathbb {N}$, suppose (45) is satisfied with

47 $$\begin{aligned} 0 \le \gamma _i^\perp \le \lambda \bar{\gamma }^\perp , \quad 0 \le \rho _i \le \lambda \bar{\rho }, \quad \text {and}\quad {\widetilde{\tau }}_0 \ge {\widetilde{\tau }}_i >0. \end{aligned}$$

If we solve (45a) exactly, define $T_i$, $\Gamma _i$, and $\Sigma _{i+1}$ through (42) and (44), and set $R_{i+1}=\rho _{i+1}I$, then the iterates of Algorithm 2 satisfy with $C_0$ and $D_{i+1}$ as in (27) the estimate

48 $$\begin{aligned}&\frac{\delta }{2}\Vert P(x^N-{\widehat{x}})\Vert ^2 + \frac{1}{\tau ^{-1}_0 + 2\gamma }\mathcal {G}^{N}\nonumber \\&\quad \le {\widetilde{\tau }}_N\tau _N\left( C_0 + \sum _{i=0}^{N-1} D_{i+1} \right) . \end{aligned}$$

If we take $\lambda =1$, then (48) holds with $\mathcal {G}^{N} = 0$.

Observe that presently

49 $$\begin{aligned} D_{i+1}= & {} {\widetilde{\tau }}_i^{-1}\gamma _i^\perp \psi ^\perp (P^\perp ({x^{i+1}-{\widehat{x}}}))\nonumber \\&+\, {\widetilde{\tau }}_{i+1}^{-1}\rho _{i+1}\phi ({y^{i+1}-{\widehat{y}}}). \end{aligned}$$

#### Proof

As we have assumed through (47), or otherwise already verified its conditions, we may apply Theorem 3.1. Multiplying (26) by ${\widetilde{\tau }}_N\tau _N$, we obtain

50 $$\begin{aligned} \frac{\delta }{2}\Vert x^N-{\widehat{x}}\Vert _P^2 + {\widetilde{q}}_N{\widetilde{\tau }}_N\tau _N \mathcal {G}^{N} \le {\widetilde{\tau }}_N\tau _N\biggl ( C_0 + \sum _{i=0}^{N-1} D_{i+1} \biggr ).\nonumber \\ \end{aligned}$$

Now, observe that solving (45a) exactly gives

51 $$\begin{aligned} {\widetilde{\tau }}_{N}^{-1}\tau _N^{-1}= & {} {\widetilde{\tau }}_{N-1}^{-1}\tau _{N-1}^{-1} + 2\gamma {\widetilde{\tau }}_{N-1}^{-1}\nonumber \\= & {} {\widetilde{\tau }}_{0}^{-1}\tau _{0}^{-1} + \sum _{j=0}^{N-1} 2\gamma {\widetilde{\tau }}_{j}^{-1} = {\widetilde{\tau }}_{0}^{-1}\tau _{0}^{-1} + 2\gamma {\widetilde{q}}_N. \end{aligned}$$

Therefore, we have the estimate

52 $$\begin{aligned} {\widetilde{q}}_N{\widetilde{\tau }}_{N}\tau _N= & {} \frac{\widetilde{q}_N}{{{\widetilde{\tau }}^{-1}_{0}}\tau _{0}^{-1}+ 2\gamma {\widetilde{q}_N}} \nonumber \\= & {} \frac{1}{{\widetilde{\tau }}_{0}^{-1}\tau _{0}^{-1} \widetilde{q}_N^{-1}+ 2\gamma } \ge \frac{1}{\tau _{0}^{-1} + 2\gamma }. \end{aligned}$$

With this, (50) yields (48).$\square$

### Primal and Dual Penalties with Projective $\Gamma$

We now study conditions that guarantee the convergence of the sum ${\widetilde{\tau }}_N\tau _N \sum _{i=0}^{N-1} D_{i+1}$ in (48). Indeed, the right-hand sides of (45b) and (45c) relate to $D_{i+1}$. In most practical cases, which we study below, $\phi$ and $\psi$ transfer these right-hand side penalties into simple linear factors within $D_{i+1}$. Optimal rates are therefore obtained by solving (45b) and (45c) as equalities, with the right-hand sides proportional to each other. Since $\eta _i \ge 0$, and it will be the case that $\eta _i=0$ for large *i*, we, however, replace (45c) by the simpler condition

53 $$\begin{aligned} \frac{1}{1-\delta } ({\widetilde{\tau }}_i^{-1} \tau _i^\perp -{\widetilde{\tau }}_{i+1}^{-1} \tau _{i+1}^\perp )\Vert K\Vert ^2 \ge -2 {\widetilde{\tau }}_{i+1}^{-1} \rho _{i+1}. \end{aligned}$$

Then, we try to make the left-hand sides of (45b) and (53) proportional with only $\tau _{i+1}^\perp$ as a free variable. That is, for some proportionality constant $\zeta > 0$, we solve

54 $$\begin{aligned} {\widetilde{\tau }}_i^{-1}\tau _i^{\perp ,-1} - {\widetilde{\tau }}_{i+1}^{-1} \tau _{i+1}^{\perp ,-1} = \zeta ({\widetilde{\tau }}_i^{-1} \tau _i^\perp -{\widetilde{\tau }}_{i+1}^{-1} \tau _{i+1}^\perp ). \end{aligned}$$

Multiplying both sides of (54) by $\zeta ^{-1}{\widetilde{\tau }}_{i+1}\tau _{i+1}^\perp$, gives on $\tau _{i+1}^\perp$ the quadratic condition

$$\begin{aligned} \tau _{i+1}^{\perp ,2} +{\widetilde{\omega }}_i(\zeta ^{-1}\tau _i^{\perp ,-1}- \tau _i^\perp ) \tau _{i+1}^\perp - \zeta ^{-1}= 0. \end{aligned}$$

Thus,

55 $$\begin{aligned} \tau _{i+1}^\perp= & {} \frac{1}{2} \left( {\widetilde{\omega }}_i(\tau _i^\perp -\zeta ^{-1}\tau _i^{\perp ,-1})\right. \nonumber \\&\left. + \sqrt{{\widetilde{\omega }}_i^2(\tau _i^\perp -\zeta ^{-1}\tau _i^{\perp ,-1})^2+4\zeta ^{-1}} \right) . \end{aligned}$$

Solving (45b) and (53) as equalities, (54) and (55) give

56 $$\begin{aligned} 2{\widetilde{\tau }}_i^{-1} \gamma _i^\perp = \frac{2\zeta (1-\delta )}{\Vert K\Vert ^2} {\widetilde{\tau }}_{i+1}^{-1} \rho _{i+1} = \zeta ({\widetilde{\tau }}_{i+1}^{-1} \tau _{i+1}^\perp -{\widetilde{\tau }}_i^{-1} \tau _i^\perp ).\nonumber \\ \end{aligned}$$

Note that this quantity is non-negative exactly when $\omega _i^\perp \ge {\widetilde{\omega }}_i$. We have

$$\begin{aligned} \frac{\omega _i^\perp }{{\widetilde{\omega }}_i}= & {} \frac{\tau _{i+1}^\perp }{\tau _i^\perp {\widetilde{\omega }}_i}\\= & {} \frac{1}{2} \left( 1-\zeta ^{-1}\tau _i^{\perp ,-2}\right. \\&\left. + \sqrt{(1-\zeta ^{-1}\tau _i^{\perp ,-2})^2+4\zeta ^{-1}{\widetilde{\omega }}_i^{-2}\tau _i^{\perp ,-2}} \right) . \end{aligned}$$

This quickly yields $\omega _i^\perp \ge {\widetilde{\omega }}_i$ if ${\widetilde{\omega }}_i \le 1$. In particular, (56) is non-negative when ${\widetilde{\omega }}_i\le 1$.

The next lemma summarises these results for the standard choice of ${\widetilde{\omega }}_i$.

#### Lemma 4.1

Let $\tau _{i+1}^\perp$ by given by (55), and set

57 $$\begin{aligned} {\widetilde{\omega }}_i=\omega _i=1/\sqrt{1+2\gamma \tau _i}. \end{aligned}$$

Then, $\omega _i^\perp \ge {\widetilde{\omega }}_i$, ${\widetilde{\tau }}_i \le {\widetilde{\tau }}_0$, and (45) is satisfied with the right-hand sides given by the non-negative quantity in (56). Moreover,

58 $$\begin{aligned} \tau _i^\perp \le \zeta ^{-1/2} \implies \tau _{i+1}^\perp \le \zeta ^{-1/2}. \end{aligned}$$

#### Proof

The choice (57) satisfies (45a), so that (45) in its entirety will be satisfied with the right-hand sides of (45b)–(45c) given by (56). The bound ${\widetilde{\tau }}_i \le {\widetilde{\tau }}_0$ follows from ${\widetilde{\omega }}_i \le 1$. Finally, the implication (58) is a simple estimation of (55).

$$\square $$

Specialisation of Algorithm 2 to the choices in Lemma 4.1 yields the steps of Algorithm 3. Observe that ${\widetilde{\tau }}_i$ entirely disappears from the algorithm. To obtain convergence rates, and to justify the initial conditions, we will shortly seek to exploit with specific $\phi$ and $\psi$ the telescoping property stemming from the non-negativity of the last term of (56).
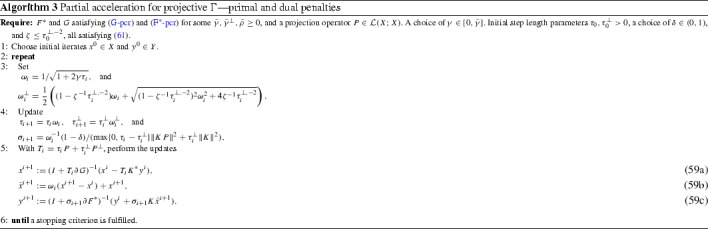


There is still, however, one matter to take care of. We need $\rho _i \le \lambda \bar{\rho }$ and $\gamma _i^\perp \le \lambda \bar{\gamma }^\perp$, although in many cases of practical interest, the upper bounds are infinite and hence inconsequential. We calculate from (55) and (57) that

60 $$\begin{aligned} \gamma _i^\perp= & {} \frac{\zeta }{2} ({\widetilde{\omega }}_i^{-1} \tau _{i+1}^\perp - \tau _i^\perp ) = \frac{1}{2} \left( -\zeta \tau _i^\perp -\tau _i^{\perp ,-1} \right. \nonumber \\&\left. +\sqrt{(\zeta \tau _i^\perp -\tau _i^{\perp ,-1})^2+4\zeta {\widetilde{\omega }}_i^{-2}} \right) \nonumber \\\le & {} \sqrt{\zeta ({\widetilde{\omega }}_i^{-2}-1)} =\sqrt{2\zeta \gamma \tau _i} \le \sqrt{2\zeta \gamma \tau _0}. \end{aligned}$$

Therefore, we need to choose $\zeta$ and $\tau _0$ to satisfy $2\zeta \gamma \tau _0 \le (\lambda \bar{\gamma }^\perp )^2$. Likewise, we calculate from (56), (57), and (60) that

$$\begin{aligned} \rho _{i+1}= & {} \frac{{\widetilde{\omega }}_i}{c} \gamma _i^\perp = \frac{\Vert K\Vert ^2 {\widetilde{\omega }}_i }{(1-\delta )\zeta } \gamma _i^\perp \le \frac{\Vert K\Vert ^2 {\widetilde{\omega }}_i }{(1-\delta )\zeta } \sqrt{2 \zeta \gamma \tau _i}\\= & {} \frac{\Vert K\Vert ^2}{(1-\delta )\zeta } \sqrt{2 \zeta \gamma \tau _0}. \end{aligned}$$

This tells us to choose $\tau _0$ and $\zeta$ to satisfy $2 \Vert K\Vert ^4/(1-\delta )^2 \zeta ^{-1} \gamma \tau _0 \le (\lambda \bar{\rho })^2$. Overall, we obtain for $\tau _0$ and $\zeta$ the condition

61 $$\begin{aligned} 0 < \tau _0 \le \frac{\lambda ^2}{2\gamma } \min \left\{ \frac{\bar{\gamma }^{\perp ,2}}{\zeta }, \frac{\bar{\rho }^2 \zeta (1-\delta )^2}{\Vert K\Vert ^4} \right\} . \end{aligned}$$

This can always be satisfied through suitable choices of $\tau _0$ and $\zeta$.

If now $\phi \equiv C_\phi$ and $\psi \equiv C_\psi ^\perp$, using the non-negativity of (56), we calculate

62 $$\begin{aligned}&\sum _{i=0}^{N-1} {\widetilde{\tau }}^{-1}_{i+1}\rho _{i+1} \phi ({y^{i+1}-{\widehat{y}}}) =\frac{\Vert K\Vert ^2C_\phi }{2(1-\delta )}\sum _{i=0}^{N-1}\nonumber \\&\quad \left( \frac{{\widetilde{\tau }}_{i+1}^{-1} \tau _{i+1}^\perp }{2} - \frac{{\widetilde{\tau }}_i^{-1} \tau _i^\perp }{2} \right) \le \frac{\Vert K\Vert ^2 C_\phi }{2(1-\delta )} {\widetilde{\tau }}_{N}^{-1} \tau _{N}^\perp . \end{aligned}$$

Similarly

63 $$\begin{aligned} \sum _{i=0}^{N-1} {\widetilde{\tau }}^{-1}_i \gamma _i^\perp \psi ({x^{i+1}-{\widehat{x}}}) \le \frac{\zeta C_\psi ^\perp }{2} {\widetilde{\tau }}_{N}^{-1} \tau _{N}^\perp . \end{aligned}$$

Using these expression to expand (49), we obtain the following convergence result.

#### Theorem 4.1

Suppose (G-pcr) and ($\hbox {F}^*$-pcr) hold for some projection operator $P \in \mathcal {L}(X; X)$, scalars $\bar{\gamma }, \bar{\gamma }^\perp , \bar{\rho }> 0$ with $\phi \equiv C_\phi$, and $\psi \equiv C_\psi ^\perp$, for some constants $C_\phi , C_\psi ^\perp >0$. With $\lambda =1/2$, fix $\gamma \in (0, \lambda \bar{\gamma }]$. Select initial $\tau _0, \tau ^\perp _0 > 0$, as well as $\delta \in (0, 1)$ and $\zeta \le (\tau _0^\perp )^{-2}$ satisfying (61). Then, Algorithm 3 satisfies for some $C_0,C_\tau >0$ the estimate

64 $$\begin{aligned}&\frac{\delta }{2}\Vert P(x^N-{\widehat{x}})\Vert ^2 + \frac{1}{\tau ^{-1}_0+2\gamma }\mathcal {G}^{N} \le \frac{C_0 C_\tau ^2}{N^2}\nonumber \\&\quad +\, \frac{C_\tau }{2 N}\left( \zeta ^{1/2} C_\psi ^\perp +\frac{\zeta ^{-1/2}\Vert K\Vert ^2}{1-\delta }C_\phi \right) , \quad (N \ge 0). \end{aligned}$$

If we take $\lambda =1$, then (48) holds with $\mathcal {G}^{N} = 0$.

#### Proof

During the course of the derivation of Algorithm 3, we have verified (45), solving (45a) as an equality. Moreover, Lemma 4.1 and (61) guarantee (47). We may therefore apply Proposition 4.1. Inserting (62) and (63) into (48) and (49) gives

65 $$\begin{aligned}&\frac{\delta }{2}\Vert P(x^N-{\widehat{x}})\Vert ^2 + \frac{1}{\tau ^{-1}_0+2\gamma }\mathcal {G}^{N} \le \tau _N{\widetilde{\tau }}_N\nonumber \\&\quad \times \, \biggl ( C_0 + \frac{\zeta C_\psi ^\perp }{2} {\widetilde{\tau }}_{N}^{-1} \tau _{N}^\perp + \frac{\Vert K\Vert ^2 C_\phi }{2(1-\delta )} {\widetilde{\tau }}_{N}^{-1} \tau _{N}^\perp \biggr ). \end{aligned}$$

The condition $\zeta \le (\tau _0^\perp )^{-2}$ now guarantees $\tau _N^\perp \le \zeta ^{-1/2}$ through (58). Now we note that ${\widetilde{\tau }}_i$ is not used in Algorithm 3, so it only affects the convergence rate estimates. We therefore simply take ${\widetilde{\tau }}_0=\tau _0$, so that ${\widetilde{\tau }}_N=\tau _N$ for all $N \in \mathbb {N}$. With this and the bound $\tau _N \le C_\tau /N$ from Remark 3.2, (64) follows by simple estimation of (65).$\square$

#### Remark 4.1

As a special case of Algorithm 3, if we choose $\zeta = \tau _0^{\perp , -2}$, then we can show from (55) that $\tau _i^\perp =\tau _0^\perp =\zeta ^{-1/2}$ for all $i \in \mathbb {N}$.

#### Remark 4.2

The convergence rate provided by Theorem 4.1 is a mixed $O(1/N^2) + O(1/N)$ rate, similarly to that derived in [5] for a type of forward–backward splitting algorithm for smooth *G*. Ours is of course backward–backward type algorithm. It is interesting to note that using the differentiability properties of infimal convolutions [23, Proposition 18.7], and the presentation of a smooth *G* as an infimal convolution, it is formally possible to derive a forward–backward algorithm from Algorithm 3. The difficulties lie in combining this conversion trick with conditions on the step lengths.

### Dual Penalty Only with Projective $\Gamma$

Continuing with the projective $\Gamma$ setup of Sect. 4.2, we now study the case $\widetilde{\mathcal {K}}=\{0\}$, that is, when only the dual penalty $\phi$ is available with $\psi \equiv 0$. To use Proposition 4.1, we need to satisfy (47) and (45), with (45a) holding as an equality. Since $\gamma _i^\perp =0$, (45b) becomes

66 $$\begin{aligned} {\widetilde{\tau }}_i^{-1}\tau _i^{\perp ,-1} - {\widetilde{\tau }}_{i+1}^{-1} \tau _{i+1}^{\perp ,-1} \ge 0. \end{aligned}$$

With respect to $\tau _{i+1}^{\perp }$, the left-hand side of (45c) is maximised (and the penalty on the right-hand side minimised) when (66) is minimised. Thus, we solve (66) exactly, which gives

$$\begin{aligned} \tau _{i+1}^{\perp }= \tau _i^{\perp }{\widetilde{\omega }}_i^{-1}. \end{aligned}$$

In consequence $\omega _i^\perp ={\widetilde{\omega }}_i^{-1}$, and (45c) becomes

67 $$\begin{aligned} \frac{1}{1-\delta }\eta _i \Vert KP\Vert ^2 + \frac{{\widetilde{\tau }}_i^{-2}}{1-\delta } (1-{\widetilde{\omega }}_{i}^{-2})\Vert K\Vert ^2 \ge -2 {\widetilde{\tau }}_{i+1}^{-1} \rho _{i+1}.\nonumber \\ \end{aligned}$$

In order to simultaneously satisfy (45a), this suggests for some, yet undetermined, $a_i>0$, to choose

68 $$\begin{aligned} {\widetilde{\omega }}_{i} :=\frac{1}{\sqrt{1+a_i{\widetilde{\tau }}_i^2}} \quad \text {and}\quad \omega _i :=\frac{1}{{\widetilde{\omega }}_i(1+2\gamma \tau _i)}. \end{aligned}$$

Since $\eta _i \ge 0$, (67) is satisfied with the choice (68) if we take

$$\begin{aligned} \rho _{i+1} = {\widetilde{\tau }}_{i+1} a_i\frac{\Vert K\Vert ^2}{2(1-\delta )}. \end{aligned}$$

To use Proposition 4.1, we need to satisfy $\rho _{i+1} \le \lambda \bar{\rho }$. Since (68) implies that $\{{\widetilde{\tau }}_i\}_{i=0}^\infty$ is non-increasing, we can satisfy this for large enough *i* if $a_i \searrow 0$. To ensure satisfaction for all $i \in \mathbb {N}$, it suffices to take $\{a_i\}_{i=0}^\infty$ non-increasing, and satisfy the initial condition

69 $$\begin{aligned} a_0 {\widetilde{\tau }}_0 \frac{\Vert K\Vert ^2}{2(1-\delta )} \le \lambda \bar{\rho }. \end{aligned}$$

The rule ${\widetilde{\tau }}_{i+1}={\widetilde{\omega }}_i{\widetilde{\tau }}_i$ and (68) give ${\widetilde{\tau }}_{i+1}^{-2} = {\widetilde{\tau }}_{i}^{-2} + a_i$. We therefore see that

$$\begin{aligned} {\widetilde{\tau }}_N^{-1}\tau _N^{-1}= & {} {\widetilde{\tau }}_0^{-1}\tau _0^{-1} + 2\gamma \sum _{i=0}^{N-1} \sqrt{\textstyle {\widetilde{\tau }}_0^{-2} + \sum _{j=0}^{i-1} a_j}\\\ge & {} 2\gamma \sum _{i=0}^{N-1} \sqrt{\textstyle {\widetilde{\tau }}_0^{-2} + \sum _{j=0}^{i-1} a_j} =: 1/\mu _0^N. \end{aligned}$$

Assuming $\phi$ to have the structure (46), moreover,

$$\begin{aligned} \sum _{i=0}^{N-1} D_{i+1}= & {} \sum _{i=0}^{N-1} \phi _{{\widetilde{\tau }}_{i+1}^{-1} R_{i+1}}({y^{i+1}-{\widehat{y}}})\nonumber \\= & {} \frac{\Vert K\Vert ^2}{2(1-\delta )} \sum _{i=0}^{N-1} a_i \phi ({y^{i+1}-{\widehat{y}}}). \end{aligned}$$

Thus, the rate (48) in Proposition 4.1 states

70 $$\begin{aligned} \frac{\delta }{2}\Vert P(x^N-{\widehat{x}})\Vert ^2 + \frac{1}{\tau ^{-1}_0 + 2\gamma }\mathcal {G}^{N} \le \mu _0^N C_0 + \frac{\Vert K\Vert ^2}{2(1-\delta )} \mu _1^N\nonumber \\ \end{aligned}$$

for

$$\begin{aligned} \mu _1^N :=\mu _0^N \sum _{i=0}^{N-1} a_i \phi ({y^{i+1}-{\widehat{y}}}). \end{aligned}$$

The convergence rate is thus completely determined by $\mu _0^N$ and $\mu _1^N$.

#### Remark 4.3

If $\phi \equiv 0$, that is, if $F^*$ is strongly convex, we may simply pick ${\widetilde{\omega }}_i=\omega _i=1/\sqrt{1+2\gamma \tau _i}$, that is $a_i=2\gamma$, and obtain from (70) a $O(1/N^2)$ convergence rate.

For a more generally applicable algorithm, suppose $\phi ({y^{i+1}-{\widehat{y}}}) \equiv C_\phi$ as in Theorem 4.1. We need to choose $a_i$. One possibility is to pick some $q \in (0, 1]$ and

71 $$\begin{aligned} a_i :={\widetilde{\tau }}_0^{-2}\bigl ((i+1)^{q}-i^{q}\bigr ). \end{aligned}$$

The concavity of $i \mapsto q^i$ for $q \in (0, 1]$ easily shows that $\{a_i\}_{i=0}^\infty$ is non-increasing. With the choice (71), we then compute

$$\begin{aligned}&\sum _{i=0}^{N-1} \sqrt{\textstyle {\widetilde{\tau }}_0^{-2} + \sum _{j=0}^{i-1} a_j} ={\widetilde{\tau }}_0^{-1}\sum _{i=0}^{N-1} i^{q/2}\nonumber \\&\quad \ge {\widetilde{\tau }}_0^{-1} \int _0^{N-1} x^{q/2} \,dx = \frac{{\widetilde{\tau }}_0^{-1}}{1+q/2}(N-1)^{1+q/2}, \end{aligned}$$

and

$$\begin{aligned} \sum _{i=0}^{N-1} a_i \le {\widetilde{\tau }}_0^{-2} N^q. \end{aligned}$$

If $N \ge 2$, we find with $C_a=(1+q/2)/(2^{1+q/2}\lambda \gamma )$ that

72 $$\begin{aligned} \mu _0^N \le \frac{{\widetilde{\tau }}_0 C_a}{N^{1+q/2}}, \quad \text {and}\quad \mu _1^N \le \frac{C_a C_\phi }{{\widetilde{\tau }}_0N^{1-q/2}}. \end{aligned}$$

The choice $q=0$ gives uniform *O*(1 / *N*) over both the initialisation and the dual sequence. By choosing $q=1$, we get $O(1/N^{3/2})$ convergence with respect to the initialisation, and $O(1/N^{1/2})$ with respect to the residual sequence.

With these choices, Algorithm 2 yields Algorithm 4, whose convergence properties are stated in the next theorem.
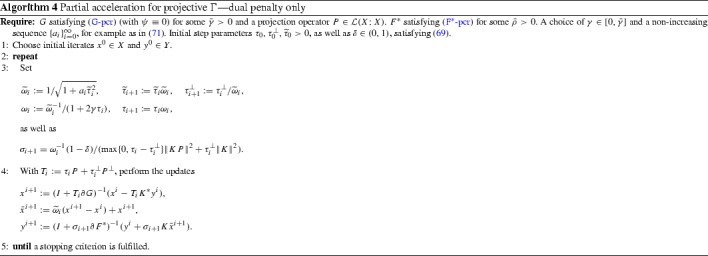


#### Theorem 4.2

Suppose (G-pcr) and ($\hbox {F}^*$-pcr) hold for some projection operator $P \in \mathcal {L}(X; X)$ and $\bar{\gamma }, \bar{\gamma }^\perp , \bar{\rho }\ge 0$ with $\psi \equiv 0$ and $\phi \equiv C_\phi$ for some constant $C_\phi \ge 0$. With $\lambda =1/2$, choose $\gamma \in (0, \lambda \bar{\gamma }]$, and pick the sequence $\{a_i\}_{i=0}^\infty$ by (71) for some $q \in (0, 1]$. Select initial $\tau _0, \tau _0^\perp , {\widetilde{\tau }}_0 >0$ and $\delta \in (0, 1)$ verifying (69). Then, Algorithm 4 satisfies

74 $$\begin{aligned}&\frac{\delta }{2}\Vert P(x^N-{\widehat{x}})\Vert ^2 + \frac{1}{\tau ^{-1}_0 + \gamma }\mathcal {G}^{N} \le \frac{{\widetilde{\tau }}_0 C_a C_0}{N^{1+q/2}}\nonumber \\&\quad + \frac{C_a C_\phi \Vert K\Vert ^2}{2(1-\delta ){\widetilde{\tau }}_0^2 N^{1-q/2}}, \quad (N \ge 2). \end{aligned}$$

If we take $\lambda =1$, then (74) holds with $\mathcal {G}^{N} = 0$.

#### Proof

We apply Proposition 4.1 whose assumptions we have verified during the course of the present section. In particular, ${\widetilde{\tau }}_i \le {\widetilde{\tau }}_0$ through the choice (68) that forces ${\widetilde{\omega }}_i \le 1$. Also, have already derived the rate (70) from (48). Inserting (72) into (70), noting that the former is only valid for $N \ge 2$, immediately gives (74).$\square$

## Examples from Image Processing and the Data Sciences

We now consider several applications of our algorithms. We generally have to consider discretisations, since many interesting infinite-dimensional problems necessitate Banach spaces. Using Bregman distances, it would be possible to generalise our work form Hilbert spaces to Banach spaces, as was done in [24] for the original method of [3]. This is, however, outside the scope of the present work.

**Fig. 1:**
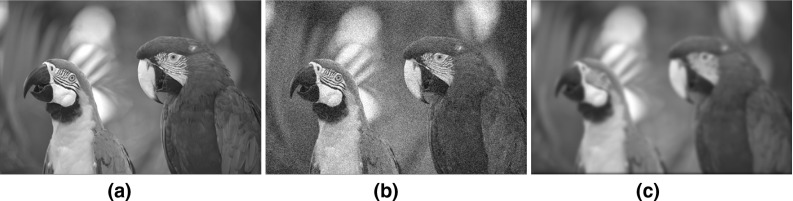
We use sample image (**b**) for denoising, and (**c**) for deblurring experiments. Free Kodak image suite photo, at the time of writing online at http://r0k.us/graphics/kodak/. **a** True image. **b** Noise image. **c** Blurry image

### Regularised Least Squares

A large range of interesting application problems can be written in the *Tikhonov regularisation* or *empirical loss minimisation* form

75 $$\begin{aligned} \min _{x \in X}~ G_0(f-Ax) + \alpha F(Kx). \end{aligned}$$

Here $\alpha >0$ is a regularisation parameter, $G_0: Z \rightarrow \mathbb {R}$ typically convex and smooth fidelity term with data $f \in Z$. The forward operator $A \in \mathcal {L}(X; Z)$—which can often also be data—maps our unknown to the space of data. The operator $K \in \mathcal {L}(X; Y)$ and the typically non-smooth and convex $F: Y \rightarrow \overline{\mathbb {R}}$ act as a regulariser.

We are particularly interested in strongly convex $G_0$ and *A* with a non-trivial null-space. Examples include, for example, Lasso—a type of regularised regression—with $G_0=\Vert x\Vert _2^2/2$, $K=I$, and $F(x)=\Vert x\Vert _1$, on finite-dimensional spaces. If the data of the Lasso is ‘sparse’, in the sense that *A* has a non-trivial null-space, then, based on accelerating the strongly convex part of the variable, our algorithm can provide improved convergence rates compared to standard non-accelerated methods.

In image processing examples abound, we refer to [25] for an overview. In total variation ($$TV $$) regularisation, we still take $$F(x)=\Vert x\Vert _1$$, but is $$K=\nabla $$ the gradient operator. Strictly speaking, this has to be formulated in the Banach space $$BV (\Omega )$$, but we will consider the discretised setting to avoid this problem. For denoising of Gaussian noise with $$TV $$ regularisation, we take $$A=I$$, and again $$G_0=\Vert x\Vert _2^2/2$$. This problem is not so interesting to us, as it is fully strongly convex. In a simple form of $$TV $$ inpainting—filling in missing regions of an image—we take *A* as a subsampling operator *S* mapping an image $$x \in L^2(\Omega )$$ to one in $$L^2(\Omega \setminus \Omega _d)$$, for $$\Omega _d \subset \Omega $$ the defect region that we want to recreate. Observe that in this case, $$\Gamma =S^*S$$ is directly a projection operator. This is therefore a problem for our algorithms! Related problems include reconstruction from subsampled magnetic resonance imaging (MRI) data (see, for example, [11, 26]), where we take $$A=S\mathfrak {F}$$ for $$\mathfrak {F}$$ the Fourier transform. Still, $$A^*A$$ is a projection operator, so the problem perfectly suits our algorithms.

Another related problem is total variation deblurring, where *A* is a convolution kernel. This problem is slightly more complicated to handle, as $A^*A$ is not a projection operator. Assuming periodic boundary conditions on a box $\Omega =\prod _{i=1}^m [c_i, d_i]$, we can write $A=\mathfrak {F}^* {\hat{a}} \mathfrak {F}$, multiplying the Fourier transform by some ${\hat{a}} \in L^2(\Omega )$. If $|{\hat{a}}| \ge \gamma$ on a subdomain, we obtain a projection form $\Gamma$ (it would also be possible to extend our theory to non-constant $\gamma$, but we have decided not to extend the length of the paper by doing so. Dualisation likewise provides a further alternative).

*Satisfaction of convexity conditions*

In all of the above examples, when written in the saddle point form (P), $F^*$ is a simple pointwise ball constraint. Lemma 2.1 thus guarantees ($\hbox {F}^*$-pcr). If $F(x)=\Vert x\Vert _1$ and $K=I$, then clearly $\Vert P^\perp {\hat{x}}\Vert$ can be bounded in $Z = L^1$ for ${\hat{x}}$ the optimal solution to (75). Thus, for some $M>0$, we can add to (75) the artificial constraint

76 $$\begin{aligned} G'(x) :=\iota _{\Vert \,\varvec{\cdot }\,\Vert _{Z} \le M}(P^\perp x). \end{aligned}$$

In finite dimensions, this gives a bound in $L^2$. Lemma 2.1 gives (G-pcr) with $\bar{\gamma }^\perp =\infty$.

In case of our total variation examples, $F(x)=\Vert x\Vert _1$ and $K=\nabla$. Provided mean-zero functions are not in the kernel of *A*, one can through Poincar’s inequality [27] on $BV (\Omega )$ and a two-dimensional connected domain $\Omega \subset \mathbb {R}^2$ show that even the original infinite-dimensional problems have bounded solutions in $L^2(\Omega )$. We may therefore again add the artificial constraint (76) with $Z=L^2$ to (75).

*Dynamic bounds and pseudo-duality gaps*

We seldom know the exact bound *M*, but can derive conservative estimates. Nevertheless, adding such a bound to Algorithm 4 is a simple, easily implemented projection of $P^\perp (x^i - T_i K^* y^i)$ into the constraint set. In practise, we do not use or need the projection, and update the bound *M* dynamically so as to ensure that the constraint (76) is never active. Indeed, *A* having a non-trivial nullspace also causes duality gaps for (P) to be numerically infinite. In [28], a ‘pseudo-duality gap’ was therefore introduced, based on dynamically updating *M*. We will also use this type of dynamic duality gaps in our reporting.

### $TGV ^2$ Regularised Problems

So far, we have considered very simple regularisation terms. Total generalised variation, $$TGV $$, was introduced in [29] as a higher-order generalisation of $$TV $$. It avoids the unfortunate stair-casing effect of $$TV $$—large flat areas with sharp transitions—while preserving the critical edge preservation property that smooth regularisers lack. We concentrate on the second-order $$TGV ^2$$. In all of our image processing examples, we can replace $$TV $$ by $$TGV ^2$$.

**Fig. 2:**
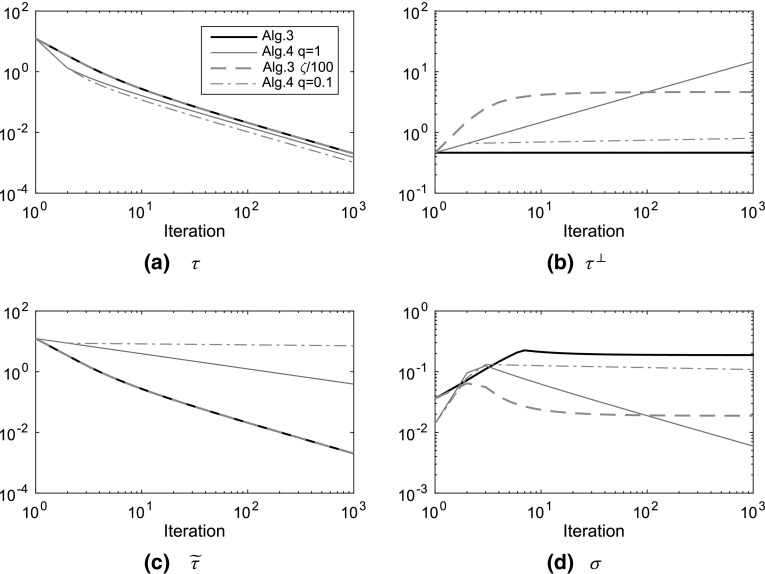
Step length parameter evolution, both axes logarithmic. ‘Alg.3’ and ‘Alg.4 q=1’ have the same parameters as our numerical experiments for the respective algorithms, in particular $\zeta =\tau _0^{\perp ,-2}$ for Algorithm 3, which yields constant $\tau ^\perp$. ‘Alg.3 $\zeta /100$’ uses the value $\zeta =\tau _0^{\perp ,-2}/100$, which causes $\tau ^\perp$ to increase for some iterations. ‘Alg.4 q=0.1’ uses the value $q=0.1$ for Algorithm 4, everything else being kept equal

As with total variation, we have to consider discretised models due the original problem being set in the Banach space $BV (\Omega )$. For two parameters $\alpha ,\beta >0$, the regularisation functional is written in the differentiation cascade form of [30] as

$$\begin{aligned} TGV ^2_{(\beta ,\alpha )}(u) :=\min _w~ \alpha \Vert \nabla u-w\Vert _1 + \beta \Vert \mathcal {E}{u}\Vert _1. \end{aligned}$$

Here $\mathcal {E}=(\nabla ^T+\nabla )/2$ is the symmetrised gradient. With $x=(v, w)$ and $y=(y_1, y_2)$, we may write the problem

77 $$\begin{aligned} \min _v G_0(f-Av) + TGV ^2_{(\beta ,\alpha )}(v), \end{aligned}$$

in the saddle point form (P) with

$$\begin{aligned} G(x):= & {} G_0(f-Av), \\ F^*(y)= & {} \iota _{\Vert \,\varvec{\cdot }\,\Vert _{L^\infty } \le \alpha }(y_1) + \iota _{\Vert \,\varvec{\cdot }\,\Vert _{L^\infty } \le \beta }(y_2),\quad \text {and}\\ K:= & {} \begin{pmatrix} \nabla &{}\quad -I \\ 0 &{}\quad \mathcal {E}\end{pmatrix}. \end{aligned}$$

If $A=I$, as is the case for denoising, we have

$$\begin{aligned} \Gamma =\gamma P \quad \text {for}\quad P=\begin{pmatrix} I &{}\quad 0 \\ 0 &{}\quad 0 \end{pmatrix}, \end{aligned}$$

perfectly uncoupling in both Algorithm 3 and Algorithm 4 the prox updates for *G* into ones for $G_1$ and $G_2$. The condition ($\hbox {F}^*$-pcr) with $\bar{\rho }=\infty$ is then immediate from Lemma 2.1. Moreover, the Sobolev–Korn inequality [31] allows us to bound on a connected domain $\Omega \subset \mathbb {R}^2$ an optimal ${\hat{w}}$ to (77) as

$$\begin{aligned} \inf _{\bar{w} \text { affine}} \Vert {\hat{w}}-\bar{w}\Vert _{L^2} \le C_\Omega \Vert \mathcal {E}{\hat{w}}\Vert _1 \le C_\Omega G_0(f) \end{aligned}$$

for some constant $C_\Omega > 0$. We may assume that $\bar{w}=0$, as the affine part of *w* is not used in (77). Therefore we may again replace $G_2=0$ by the artificial constraint $G_2(w)=\iota _{\Vert \,\varvec{\cdot }\,\Vert _{L^2} \le M}(w)$. By Lemma 2.1, *G* will then satisfy (G-pcr) with $\bar{\gamma }^\perp =\infty$.

### Numerical Results

We demonstrate our algorithms on $$TGV ^2$$ denoising and $$TV $$ deblurring. Our tests are done on the photographs in Fig. 1, both at the original resolution of $$768 \times 512$$, and scaled down by a factor of 0.25 to $$192 \times 128$$ pixels. It is image #23 from the free Kodak image suite. Other images from the collection that we have experimented on give analogous computational results. For both of our example problems, we calculate a target solution by taking one million iterations of the basic PDHGM (3). We also tried interior point methods for this, but they are only practical for the smaller denoising problem.

We evaluate Algorithms 3 and 4 against the standard unaccelerated PDHGM of [3], as well as (a) the mixed-rate method of [5], denoted here C-L-O, (b) the relaxed PDHGM of [20, 32], denoted here ‘Relax’, and (c) the adaptive PDHGM of [33], denoted here ‘Adapt’. All of these methods are very closely linked and have comparable low costs for each step. This makes them straightforward to compare.

As we have discussed, for comparison and stopping purposes, we need to calculate a pseudo-duality gap as in [28], because the real duality gap is in practise infinite when *A* has a non-trivial nullspace. We do this dynamically; upgrading, the *M* in (76) every time, we compute the duality gap. For both of our example problems, we use for simplicity $Z=L^2$ in (76). In the calculation of the final duality gaps comparing each algorithm, we then take as *M* the maximum over all evaluations of all the algorithms. This makes the results fully comparable. We always report the duality gap in decibels $10\log _{10}(\text {gap}^2/\text {gap}_0^2)$ relative to the initial iterate. Similarly, we report the distance to the target solution ${\hat{u}}$ in decibels $10\log _{10}(\Vert u^i-{\hat{u}}\Vert ^2/\Vert {\hat{u}}\Vert ^2)$, and the primal objective value $\text {val}(x) :=G(x)+F(Kx)$ relative to the target as $10\log _{10}(\text {val}(x)^2/\text {val}({\hat{x}})^2)$. Our computations were performed in MATLAB+C-MEX on a MacBook Pro with 16GB RAM and a 2.8 GHz Intel Core i5 CPU.

$TGV ^2$ *denoising* The noise in our high-resolution test image, with values in the range [0, 255], has standard deviation 29.6 or 12 dB. In the downscaled image, these become, respectively, 6.15 or 25.7 dB. As parameters $(\beta , \alpha )$ of the $TGV ^2$ regularisation functional, we choose (4.4, 4) for the downscale image, and translate this to the original image by multiplying by the scaling vector $(0.25^{-2}, 0.25^{-1})$ corresponding to the 0.25 downscaling factor. See [34] for a discussion about rescaling and regularisation factors, as well as for a justification of the $\beta /\alpha$ ratio.

**Fig. 3:**
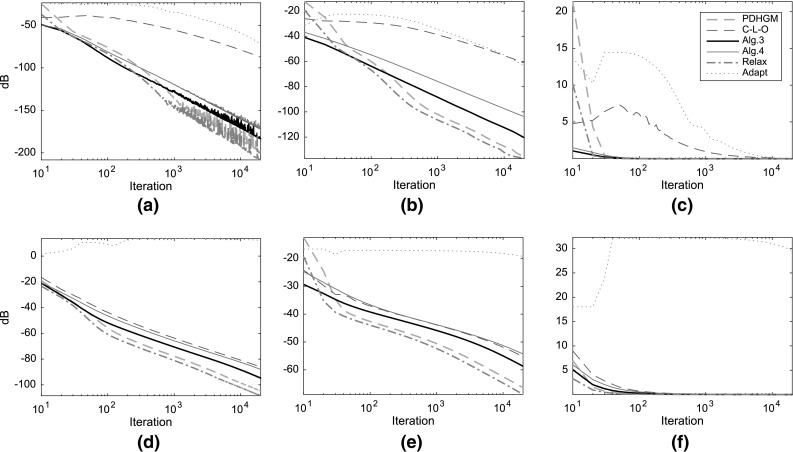
$TGV ^2$ denoising performance, 20,000 iterations, high- and low-resolution images. The plot is logarithmic, with the decibels calculated as in Sect. 5.3. The poor high-resolution results for ‘Adapt’ [33] have been omitted to avoid poor scaling of the plots. **a** Gap, low resolution, **b** target, low resolution, **c** value, low resolution, **d** gap, high resolution, **e** target, high resolution, **f** value, high resolution

For the PDHGM and our algorithms, we take $\gamma =0.5$, corresponding to the gap convergence results. We choose $\delta =0.01$, and parametrise the PDHGM with $\sigma _0=1.9/\Vert K\Vert$ and $\tau _0^*=\tau _0 \approx 0.52/\Vert K\Vert$ solved from $\tau _0\sigma _0=(1-\delta )\Vert K\Vert ^2$. These are values that typically work well. For forward-differences discretisation of $TGV ^2$ with cell width $h=1$, we have $\Vert K\Vert ^2 \le 11.4$ [28]. We use the same value of $\delta$ for Algorithm 3 and Algorithm 4, but choose $\tau _0^\perp = 3\tau _0^*$, and $\tau _0={\widetilde{\tau }}_0=80\tau _0^*$. We also take $\zeta =\tau _0^{\perp ,-2}$ for Algorithm 3. These values have been found to work well by trial and error, while keeping $\delta$ comparable to the PDHGM. A similar choice of $\tau _0$ with a corresponding modification of $\sigma _0$ would significantly reduce the performance of the PDHGM. For Algorithm 4, we take exponent $q=0.1$ for the sequence $\{a_i\}$. This gives in principle a mixed $O(1/N^{1.5})+O(1/N^{0.5})$ rate, possibly improved by the convergence of the dual sequence. We plot the evolution of the step length for these and some other choices in Fig. 2. For the C-L-O, we use the detailed parametrisation from [35, Corollary 2.4], taking as $\Omega _Y$ the true $L^2$-norm Bregman divergence of $B(0, \alpha ) \times B(0, \beta )$, and $\Omega _X=10 \cdot \Vert f\Vert ^2/2$ as a conservative estimate of a ball containing the true solution. For ‘Adapt’, we use the exact choices of $\alpha _0$, $\eta$, and *c* from [33]. For ‘Relax’, we use the value 1.5 for the inertial $\rho$ parameter of [32]. For both of these algorithms, we use the same choices of $\sigma _0$ and $\tau _0$ as for the PDHGM.

**Table 1:** $TGV ^2$ denoising performance, maximum 20,000 iterations

Low resolution						
Method	Gap $\le -50$ dB		Tgt $\le -40$ dB		Val $\le 1$ dB	
	Iter	Time (s)	Iter	Time (s)	Iter	Time (s)
PDHGM	30	0.40	40	0.46	30	0.40
C-L-O	500	4.67	1210	11.31	970	9.04
Alg.3	20	0.29	10	0.22	20	0.29
Alg.4	20	0.47	20	0.47	20	0.47
Relax	20	0.34	30	0.45	20	0.34
Adapt	5360	106.63	2040	41.38	3530	70.78

High resolution						
Method	Gap $\le -40$ dB		Tgt $\le -30$ dB		Val $\le 1$ dB	
	Iter	Time (s)	Iter	Time (s)	Iter	Time (s)
PDHGM	50	8.85	30	5.13	30	5.13
C-L-O	80	15.76	30	5.97	80	15.76
Alg.3	40	6.20	20	3.10	40	6.20
Alg.4	60	9.18	30	4.53	60	9.18
Relax	40	7.45	20	3.70	20	3.70
Adapt	$\textendash$	$\textendash$	$\textendash$	$\textendash$	$\textendash$	$\textendash$

The CPU time and number of iterations (at a resolution of 10) needed to reach given solution quality in terms of the duality gap, distance to target, or primal objective value

**Fig. 4:**
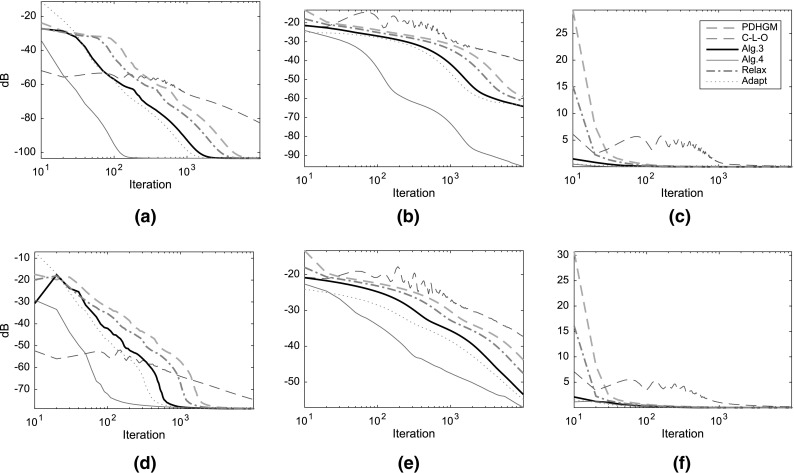
$$TV $$ deblurring performance, 10,000 iterations, high- and low-resolution images. The plot is logarithmic, with the decibels calculated as in Sect. 5.3. **a** Gap, low resolution. **b** Target, low resolution. **c** Value, low resolution. **d** Gap, high resolution. **e** Target, high resolution. **f** Value, high resolution

We take fixed 20,000 iterations and initialise each algorithm with $y^0=0$ and $x^0=0$. To reduce computational overheads, we compute the duality gap and distance to target only every 10 iterations instead of at each iteration. The results are in Fig. 3 and Table 1. As we can see, Algorithm 3 performs extremely well for the low-resolution image, especially in its initial iterations. After about 700 or 200 iterations, depending on the criterion, the standard and relaxed PDHGM start to overtake. This is a general effect that we have seen in our tests: the standard PDHGM performs in practise very well asymptotically, although in principle all that exists is a *O*(1 / *N*) rate on the ergodic duality gap. Algorithm 4, by contrast, does not perform asymptotically so well. It can be extremely fast on its initial iterations, but then quickly flattens out. The C-L-O surprisingly performs better on the high-resolution image than on the low-resolution image, where it does somewhat poorly in comparison with the other algorithms. The adaptive PDHGM performs very poorly for $TGV ^2$ denoising, and we have indeed excluded the high-resolution results from our reports to keep the scaling of the plots informative. Overall, Algorithm 3 gives good results fast, although the basic and relaxed PDHGM seems to perform, in practise, better asymptotically.

$$TV $$ *deblurring* Our test image has now been distorted by Gaussian blur of standard deviation 4, which we intent to remove. We denote by $${\hat{a}}$$ the Fourier presentation of the blur operator as discussed in Sect. 5.1. For numerical stability of the pseudo-duality gap, we zero out small entries, replacing this $${\hat{a}}$$ by $${\hat{a}} \chi _{|{\hat{a}}(\,\varvec{\cdot }\,)| \ge \Vert {\hat{a}}\Vert _\infty /1000}(\xi )$$. Note that this is only needed for the stable computation of $$G^*$$ for the pseudo-duality gap, to compare the algorithms; the algorithms themselves are stable without this modification. To construct the projection operator *P*, we then set $${\hat{p}}(\xi )=\chi _{|{\hat{a}}(\,\varvec{\cdot }\,)| \ge 0.3 \Vert {\hat{a}}\Vert _\infty }(\xi )$$, and $$P=\mathfrak {F}^* {\hat{p}} \mathfrak {F}$$.

**Table 2:** $$TV $$ deblurring performance, maximum 10,000 iterations

Method	Low resolution						High resolution					
	Gap $\le -60$ dB		Tgt $\le -40$ dB		Val $\le 1$ dB		Gap $\le -60$ dB		Tgt $\le -30$ dB		Val $\le 1$ dB	
	Iter	Time (s)	Iter	Time (s)	Iter	Time (s)	Iter	Time (s)	Iter	Time (s)	Iter	Time (s)
PDHGM	390	2.53	2630	17.41	60	0.47	1180	118.30	970	98.98	70	6.59
C-L-O	600	3.81	8930	54.20	950	5.95	500	48.44	1940	187.42	1000	96.60
Alg.3	130	1.14	880	7.22	20	0.25	400	58.42	320	46.16	40	6.13
Alg.4	30	0.47	90	0.97	10	0.29	60	7.97	50	6.66	30	3.98
Relax	260	1.62	1750	11.34	40	0.29	790	77.31	650	63.84	50	5.29
Adapt	110	1.12	660	5.94	10	0.16	260	39.39	150	23.30	30	4.72

The CPU time and number of iterations (at a resolution of 10) needed to reach given solution quality in terms of the duality gap, distance to target, or primal objective value

We use $$TV $$ parameter 2.55 for the high-resolution image and the scaled parameter $$2.55*0.15$$ for the low-resolution image. We parametrise all the algorithms almost exactly as $$TGV ^2$$ denoising above, of course with appropriate $$\Omega _U$$ and $$\Vert K\Vert ^2 \le 8$$ corresponding to $$K=\nabla $$ [36]. The only difference in parameterisation is that we take $$q=1$$ instead of $$q=0.1$$ for Algorithm 4.

The results are in Fig. 4 and Table 2. It does not appear numerically feasible to go significantly below $-100$ or $-80$ dB gap. Our guess is that this is due to the numerical inaccuracies of the fast Fourier transform implementation in MATLAB. The C-L-O performs very well judged by the duality gap, although the images themselves and the primal objective value appear to take a little bit longer to converge. The relaxed PDHGM is again slightly improved from the standard PDHGM. The adaptive PDHGM performs very well, slightly outperforming Algorithm 3, although not Algorithm 4. This time Algorithm 4 performs remarkably well.

## Conclusion

To conclude, overall, our algorithms are very competitive within the class of proposed variants of the PDHGM. Within our analysis, we have, moreover, proposed very streamlined derivations of convergence rates for even the standard PDHGM, based on the proximal point formulation and the idea of testing. Interesting continuations of this study include whether the condition ${\hat{T}}_i K=K{\widetilde{T}}_i$ can reasonably be relaxed such that ${\hat{T}}_i$ and ${\widetilde{T}}_i$ would not have to be scalars, as well as the relation to block coordinate descent methods, in particular [14, 37].
